# Endovascular Thrombectomy for Acute Ischemic Stroke Due to Large Vessel Occlusion with Large Ischemic Core: A Systematic Review and Meta-Analysis

**DOI:** 10.3390/neurosci7050102

**Published:** 2026-09-17

**Authors:** Abdullah M. Alkutbi, Hussain Almohammed, Ahmed Salah Morad, Bushra Wadi Bin Saddiq, Kawthar Faisal Kushara, Wejdan Ahmed Aldawsari, Abdullah Ahmed Albesher, Jana M. Alsyrwan, Fatimah Ibrahim Almuhaysin, Lamyaa Mohammed Gharawi

**Affiliations:** 1 Department of Neurology, College of Medicine, King Abdulaziz University, Jeddah 21589, Saudi Arabia; 2 College of Medicine, King Faisal University, Al Ahsa 31982, Saudi Arabia; 3 Batterjee Medical College, Jeddah 21442, Saudi Arabia; 4 College of Medicine, King Abdulaziz University, Jeddah 21589, Saudi Arabia; 5 College of Medicine, Tabuk University, Tabuk 47918, Saudi Arabia

**Keywords:** endovascular thrombectomy, acute ischemic stroke, large ischemic core, large vessel occlusion, medical management

## Abstract

Background: Multiple trials have evaluated the benefits of endovascular thrombectomy (EVT) in patients with large-core ischemic stroke. This study aimed to determine the efficacy and safety of EVT compared with medical management (MM) alone in patients with large ischemic core infarcts. Methods: This study was registered in PROSPERO (CRD420261360129). A comprehensive search of PubMed, Web of Science, and the Cochrane Library was conducted to identify all relevant studies published up to April 2026. Effect sizes were calculated as risk ratios (RRs) using random-effects or fixed-effects models, as appropriate. Study quality was assessed using the Cochrane Risk of Bias tool and the Newcastle–Ottawa Scale. Subgroup and sensitivity analyses were performed to explore heterogeneity and evaluate outcomes across different patient populations. Outcomes assessed included excellent functional independence (mRS 0–2) and favorable functional outcome (mRS 0–3) at 3 months, mean mRS at 3 and 12 months, symptomatic intracranial hemorrhage, and all-cause mortality at 3 and 12 months. Results: Nine eligible studies (5 randomized controlled trials [RCTs] and 4 cohort studies; total sample size = 2252) were included to evaluate the comparative efficacy and safety of EVT versus MM alone in patients with acute ischemic stroke (AIS) secondary to large vessel occlusion. Our pooled analysis demonstrated a significantly higher rate of achieving a modified Rankin Scale (mRS) score of 0–2 in the EVT group compared with the MM group (RR = 2.64, 95% CI [1.79–3.90], *p* < 0.00001). Similarly, the likelihood of achieving an mRS score of 0–3 was significantly greater with EVT than with MM (RR = 1.78, 95% CI [1.40–2.26], *p* < 0.00001). The mean mRS score at 3 months was also significantly lower in the EVT group (MD = −0.58, 95% CI [−0.77 to −0.39], *p* < 0.00001). Safety outcomes were comparable between groups, with no significant differences in the rates of symptomatic intracranial hemorrhage (sICH) (RR = 1.36, 95% CI [0.88–2.11], *p* = 0.17) or mortality (RR = 0.96, 95% CI [0.65, 1.42], *p* = 0.84, I^2^ = 75%). Conclusions: Our findings suggest that EVT is associated with improved functional outcomes compared with MM alone, particularly in evidence derived from RCTs, while maintaining an acceptable safety profile. Further high-quality RCTs are warranted to strengthen the evidence base and validate these findings.

## 1. Introduction

Acute ischemic stroke (AIS) remains a leading cause of mortality and long-term disability worldwide, with the global burden of stroke continuing to rise despite advances in prevention and acute care [1]. Large-vessel occlusion (LVO) of the anterior circulation accounts for a disproportionate share of stroke-related morbidity and mortality because occlusion of the internal carotid artery or proximal middle cerebral artery rapidly threatens extensive territories of salvageable brain tissue [2]. Although intravenous thrombolysis is the cornerstone of acute reperfusion therapy, it achieves recanalization in fewer than 30% of proximal anterior-circulation LVOs, with particularly low rates for internal carotid artery occlusions [3].

Endovascular thrombectomy (EVT) transformed the treatment of anterior-circulation LVO after multiple landmark randomized controlled trials, and the HERMES individual-patient-data meta-analysis demonstrated substantial improvements in functional outcomes when EVT was performed within 6 h of symptom onset [4]. Subsequently, the DAWN trial extended the treatment window to 6–24 h from the last known well using a clinical-core mismatch paradigm, and the DEFUSE 3 trial extended eligibility to 6–16 h from symptom onset by selecting patients with a target perfusion-imaging mismatch profile; both approaches identified individuals with persistent salvageable tissue despite delayed presentation [5,6].

However, patients with large baseline ischemic cores were systematically excluded from these pivotal trials because of concerns about futile reperfusion, symptomatic intracranial hemorrhage, reperfusion injury, and malignant cerebral edema [7]. Across studies, large-core infarction has been defined heterogeneously—using low Alberta Stroke Program Early CT Score (ASPECTS) values on non-contrast CT, substantial ischemic-core volumes on CT perfusion (e.g., ≥50 mL or ≥70 mL), or large volumes on diffusion-weighted MRI—which has contributed to substantial variability in patient selection, trial design, and outcome assessment [8,9,10,11,12,13].

Between 2022 and 2024, several dedicated randomized trials—RESCUE-Japan LIMIT, SELECT2, ANGEL-ASPECT, TENSION, TESLA, and LASTE—specifically evaluated EVT in patients with large-core ischemic stroke [8,9,10,11,12,13]. Collectively, these studies suggested that EVT may improve functional outcomes in selected patients with extensive baseline infarction. However, treatment effects varied across imaging paradigms, infarct-core definitions, treatment windows, and reperfusion-related safety outcomes [8,9,10,11,12,13]. Important uncertainties therefore persist regarding optimal patient selection, imaging criteria, and the balance between functional benefit and hemorrhagic risk in this population [14].

Three previous systematic reviews and meta-analyses have examined EVT in large-core stroke, each with limitations that leave key questions unresolved. An early meta-analysis of studies published between 2015 and 2020 (10 studies, 954 patients) demonstrated that, among patients with a volumetrically defined large ischemic core (≥50 mL), mechanical thrombectomy was associated with a lower rate of unfavorable 90-day functional outcomes compared with medical management alone; however, that analysis predated all dedicated large-core randomized trials and was based predominantly on observational data [15]. A more recent systematic review and meta-analysis restricted to randomized trials (search through November 2023) provided high-quality evidence from six trials (*n* = 1897) that thrombectomy significantly reduced disability at 3 months (odds ratio for good functional outcome, 2.90; 95% CI, 2.08–4.05), but its literature search was completed before the publication of the TESLA and LASTE trials, and heterogeneity across imaging modalities, ASPECTS thresholds, and core-volume definitions was not systematically explored [16]. Another systematic review and meta-analysis, also with a search through November 2023, compared bridging intravenous thrombolysis plus EVT versus EVT alone in patients with ASPECTS ≤ 5 and found no significant differences in functional independence, recanalization success, or mortality; however, it addressed a distinct therapeutic question (bridging thrombolysis) rather than the fundamental efficacy of EVT versus medical management in the large-core population [17].

Consequently, an updated, comprehensive synthesis that incorporates all major randomized evidence and rigorously evaluates effect modifiers is needed. We therefore performed a systematic review and meta-analysis to determine the efficacy and safety of EVT compared with medical management in patients with AIS and a large ischemic core.

## 2. Methodology

This systematic review followed the Preferred Reporting Items for Systematic Reviews and Meta-Analyses (PRISMA) guidelines to improve the transparency and reproducibility of its methodologies [18]. The study protocol was submitted and published in the International Prospective Register of Systematic Reviews (PROSPERO) under the registration number CRD420261360129. This study did not require institutional research ethics board approval due to the nature of the study.

### 2.1. Search Strategy

Two researchers (HA and KK) conducted a comprehensive systematic search across PubMed, Web of Science, and the Cochrane Library databases to identify all relevant articles published until April 2026. The keywords included in the search strategy were as follows: (“mechanical thrombectomy” OR “endovascular thrombectomy”) AND (“acute ischemic stroke” OR “AIS”) AND (“large vessel occlusion” OR “LVO”) AND (“randomized controlled trial” OR “randomised controlled trial” OR “RCT” OR “clinical trial” OR “controlled clinical trial” OR “cohort study” OR “prospective cohort” OR “retrospective cohort” OR “observational study” OR “comparative study”).

### 2.2. Study Selection

To ensure methodological rigor, studies were selected based on predefined inclusion and exclusion criteria. We included randomized controlled trials (RCTs) and cohort studies involving adult patients (≥18 years), including elderly patients (>80 years), with AIS and a large ischemic core, defined as ASPECTS ≤ 5–6 or infarct core volume ≥ 50 mL. Eligible studies were required to compare EVT with medical management alone and report at least one relevant outcome, including modified Rankin Scale (mRS) scores, National Institutes of Health Stroke Scale (NIHSS) improvement at 24–48 h, 90-day mortality, recurrence of cerebral infarction, decompressive craniectomy, or symptomatic intracranial hemorrhage (sICH). These were the candidate outcomes used at the screening stage; of these, mRS (0–2), mRS (0–3), mean mRS, sICH, and mortality were ultimately reported with sufficient data across studies to be quantitatively synthesized, as detailed in Section 2.7. No restrictions were applied regarding imaging modality, and only full-text English-language studies were included. We excluded case reports, case–control studies, reviews, editorials, conference abstracts, studies without a control group, and studies that did not report the primary outcomes of interest.

### 2.3. Screening

Two reviewers (KK and HA) independently and simultaneously screened the studies by title and abstract using the Rayyan web tool based on predefined inclusion and exclusion criteria [19]. Studies that fulfilled the inclusion criteria underwent full-text review, which was conducted independently by KK and LA. Any disagreements were addressed and resolved through discussion among the reviewers.

### 2.4. Data Extraction

Two reviewers (JA and FA) independently performed data extraction using a piloted data extraction form developed in Microsoft Excel. Any discrepancies between the reviewers were resolved through discussion and consensus, and when necessary, consultation with a third senior reviewer was undertaken. For each eligible study, we extracted general trial characteristics, including the primary author, year of publication, trial name, and geographic location. Baseline participant characteristics were also collected to assess comparability between study groups, including inclusion and exclusion criteria, mean age, sex, and median baseline NIHSS score with interquartile range (IQR). Regarding the intervention and comparator groups, we extracted data on occlusion site, Alberta Stroke Program Early CT Score (ASPECTS) median value (IQR), median infarct-core volume (IQR) in milliliters, use of intravenous thrombolysis [no. (%)], and the interval between stroke onset and randomization [median (IQR), minutes].

### 2.5. Risk of Bias Assessment

The methodological quality of included randomized controlled trials was assessed using the Cochrane Risk of Bias 2 (RoB 2) tool [20], evaluating domains including the randomization process, deviations from intended interventions, missing outcome data, outcome measurement, and selective reporting. Observational cohort studies were assessed using the Newcastle–Ottawa Scale (NOS) [21], which evaluates study quality based on selection, comparability, and outcome assessment.

### 2.6. Certainty of Evidence Assessment

The Grading of Recommendations, Assessment, Development, and Evaluations (GRADE) approach was used to assess the certainty of the evidence. The quality of evidence was classified as high, moderate, low, or very low quality.

### 2.7. Outcome Measures

Primary efficacy outcomes were excellent functional independence (mRS 0–2), favorable functional outcome (mRS 0–3), mean mRS score at 90 days, and mean mRS score at 12 months. Secondary outcomes were the safety outcomes sICH and all-cause mortality, each assessed within 90 days and at 12 months.

### 2.8. Data Analysis

Statistical analysis was conducted using RevMan Manager (RevMan) Version 5.0 software, which aided in the pooling of the study results and evaluation of overall effects [22,23]. Heterogeneity across studies was examined using the Cochrane-based Q statistic and I^2^ test, which determined the type of model to be used in the analysis. A random-effects model was applied when substantial heterogeneity was present (*p* < 0.05, I^2^ > 50%). In cases where heterogeneity was minimal (I^2^ < 50%, *p* > 0.05), a fixed-effects model was used. Standardized mean differences (SMD) for continuous outcomes and odds ratios (OR) for dichotomous variables were used to express the location of lesions seen on MRI; each had a 95% confidence interval (CI). Statistical significance was defined as *p* < 0.05. Subgroup analyses will be performed to evaluate potential effect modifiers based on the type of study, site of occlusion, infarct core volume, follow-up duration, sex, and geographic region if feasible.

## 3. Results

### 3.1. Search Results

**Figure 1:**
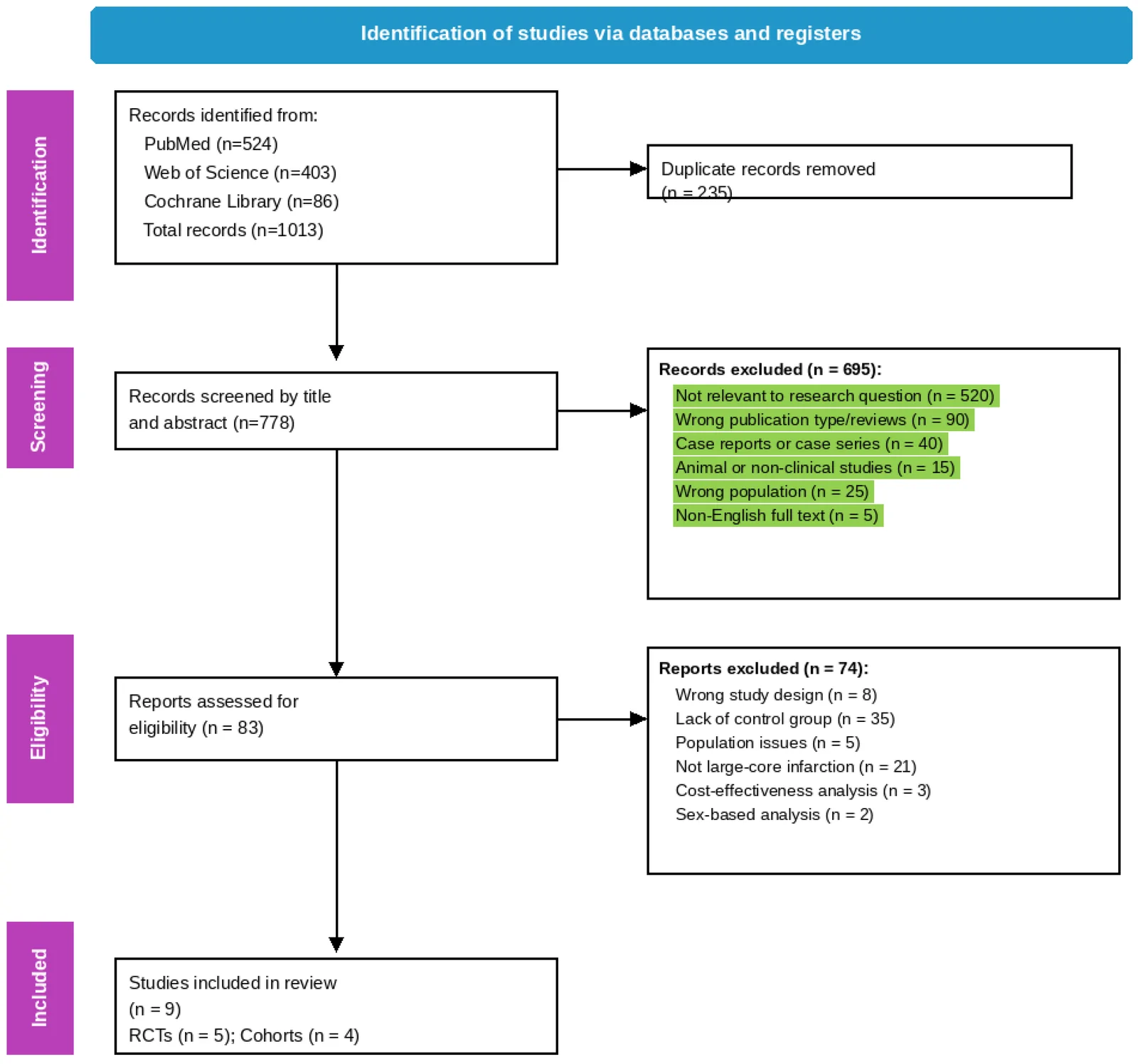
PRISMA flow chart of the included studies.

Figure 1 represents the PRISMA flowchart, which depicts the stages of study inclusion in our review. Using our search strategy, we retrieved 1013 studies from these databases: Cochrane Library, PubMed, and Web of Science. After 235 duplicates were eliminated, 778 records remained for screening. Of these, 695 records were removed because they were irrelevant to the research questions. The eligibility of the remaining 83 full-text articles was evaluated. Seventy-four reports were excluded following full-text review for the reasons detailed in Figure 1. Eventually, this systematic review and meta-analysis contained 9 papers [8,9,10,11,24,25,26,27,28].

### 3.2. Study Characteristics

Table 1 summarizes the characteristics of the included studies. Nine studies were included in our analysis, five of which were RCTs and four were retrospective studies. Four studies were conducted in China [10,24,25,27], three studies in Europe [9,11,26], and two studies in Japan [8,28]. Most of the studies had a 3-month follow-up duration, except for Huo et al. (2025) [24] and Thomalla et al. (2024) [11], which had a 12-month follow-up duration. The total number of participants in the studies was 2252 (848 females and 1247 males) with a mean age of 69.64 ± 12.21 years. We have provided more detailed information about the baseline characteristics of the included studies in Table S1.

### 3.3. Quality Assessment

**Figure 2:**
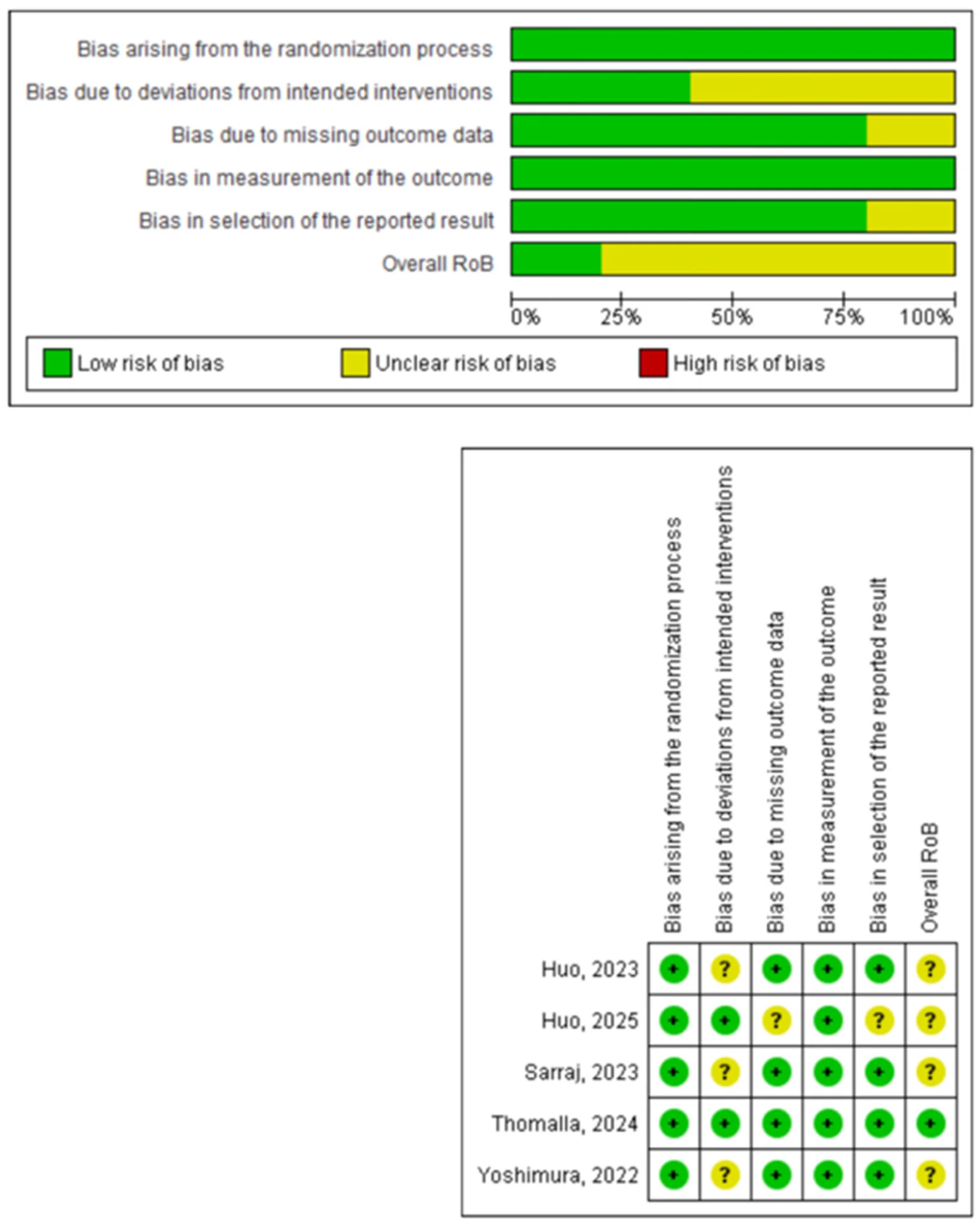
Quality assessment for RCTs [8,9,10,11,24].

The risk of bias of the included RCTs was assessed using the ROB 2 tool. Overall, most studies were judged to have some concerns, while only one study, Thomalla et al. (2024) [11], was rated as having a low risk of bias across all domains. Huo et al. (2025) [24] showed concerns in Domain 3 (missing outcome data) and Domain 5 (selection of the reported result) due to incomplete outcome data and unclear statistical analysis plans. Yoshimura et al. (2022) [8] raised concerns in Domain 2 (deviations from intended interventions), likely related to its open-label design and the potential for deviations from the study protocol. Similarly, Huo et al. (2023) [10] and Sarraj et al. (2023) [9] demonstrated concerns in Domain 2, primarily due to open-label designs that allowed treatment modifications, such as crossover between treatment groups or protocol deviations (Figure 2).

**Figure 3:**
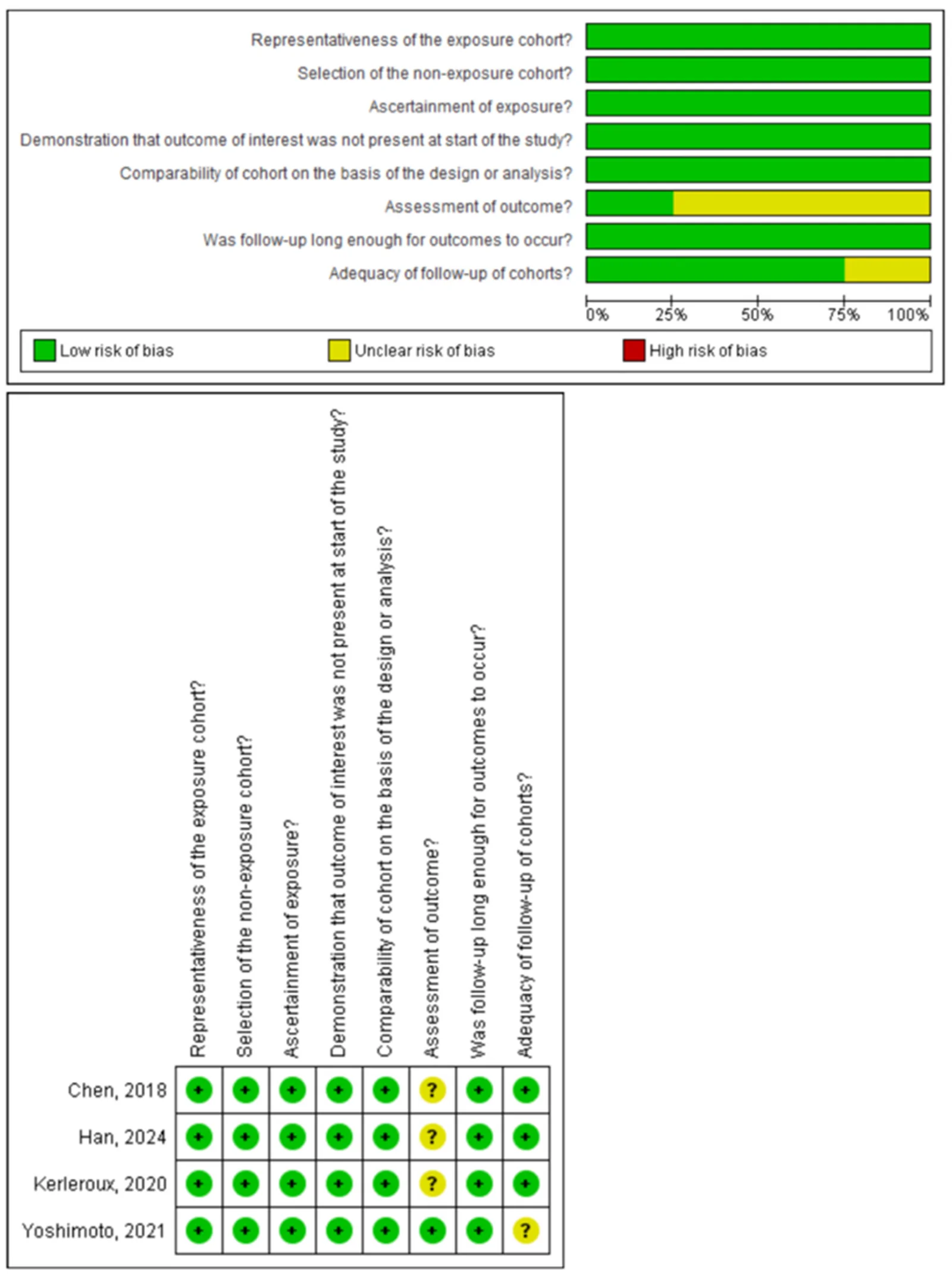
Quality assessment for cohort studies [25,26,27,28].

For the cohort studies, the Newcastle–Ottawa Scale (NOS) was used to assess the risk of bias. All included studies were rated as having a low risk of bias, with quality scores ranging from 7 to 8 stars. Han et al. (2024) [25] received 7 stars, while Chen et al. (2018) [27], Kerleroux et al. (2020) [26], and Yoshimoto et al. (2021) [28] each received 8 stars. Most studies achieved high scores across the selection, comparability, and outcome domains. Minor limitations included unclear reporting of outcome assessment or blinding procedures and incomplete follow-up reporting in one study. Overall, the methodological quality of the included cohort studies was considered good (Figure 3).

### 3.4. Certainty of Evidence

The certainty of evidence for all critical and important outcomes was assessed by the GRADE approach, as shown in Table 2. The two efficacy outcomes, mRS (0–2) and mRS (0–3), were downgraded from high to moderate certainty because of serious inconsistency (I^2^ = 67% and 66%, respectively, driven largely by the cohort studies, in which several trials were rated as having “some concerns” on RoB 2); the remaining outcomes (mean mRS, sICH, and mortality), which showed low heterogeneity (I^2^ ≤ 34%), retained a high certainty rating. When stratified by follow-up duration (Figures 6 and 8), mean mRS at 3 months showed substantial heterogeneity (I^2^ = 61%) and was downgraded one level to moderate certainty, whereas mean mRS at 12 months (I^2^ = 0%) retained high certainty; mortality at 3 months (I^2^ = 0%) retained high certainty, whereas mortality at 12 months showed substantial heterogeneity (I^2^ = 75%) with only two contributing studies and was downgraded one level to moderate certainty for inconsistency and imprecision.

### 3.5. Outcomes

#### 3.5.1. Summary of Outcomes

Table 3 summarizes the outcomes of the meta-analysis related to the efficacy and safety of EVT. Primary outcomes included favorable functional outcome (mRS 0–3), excellent functional outcome (mRS 0–2), and mean mRS. Secondary outcomes included safety profiles, sICH, and all-cause mortality rates.

#### 3.5.2. Efficacy Outcomes

**Figure 4:**
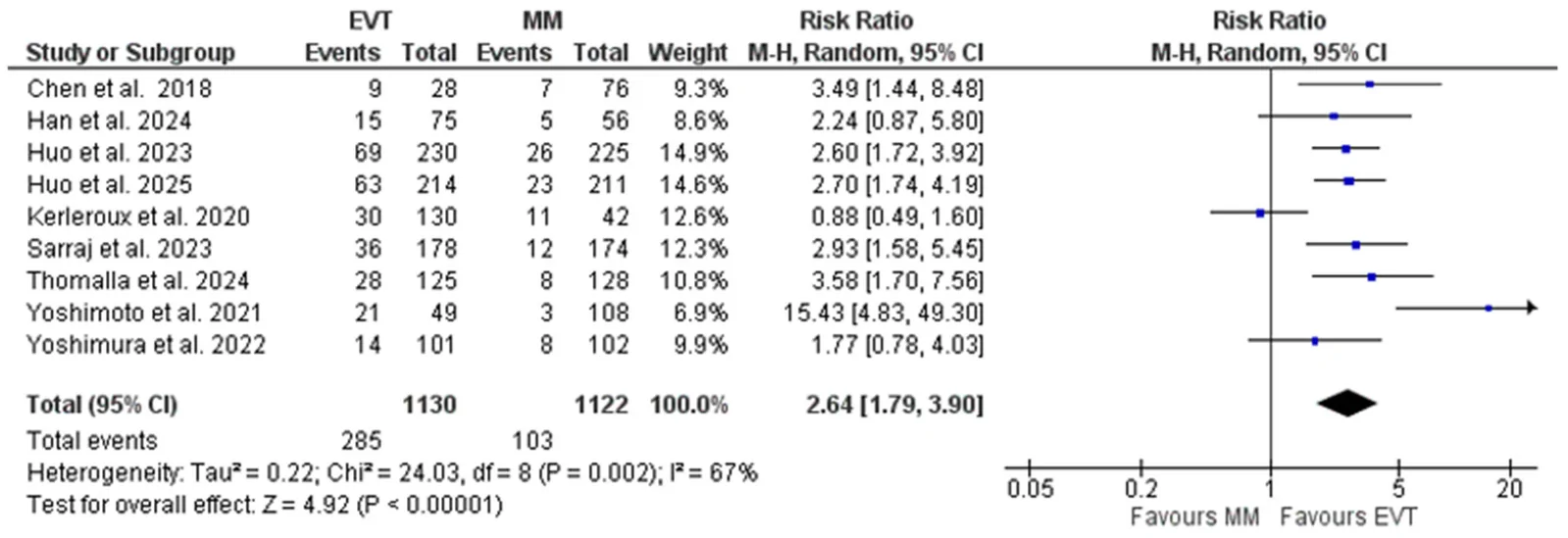
RR of mRS (0–2) [8,9,10,11,24,25,26,27,28].

The pooled analysis showed a significantly increased rate of achieving an mRS score of (0–2) at 3 months, favoring EVT when compared to MM (Figure 4, RR = 2.64, 95% CI [1.79, 3.90], *p* < 0.00001).

**Figure 5:**
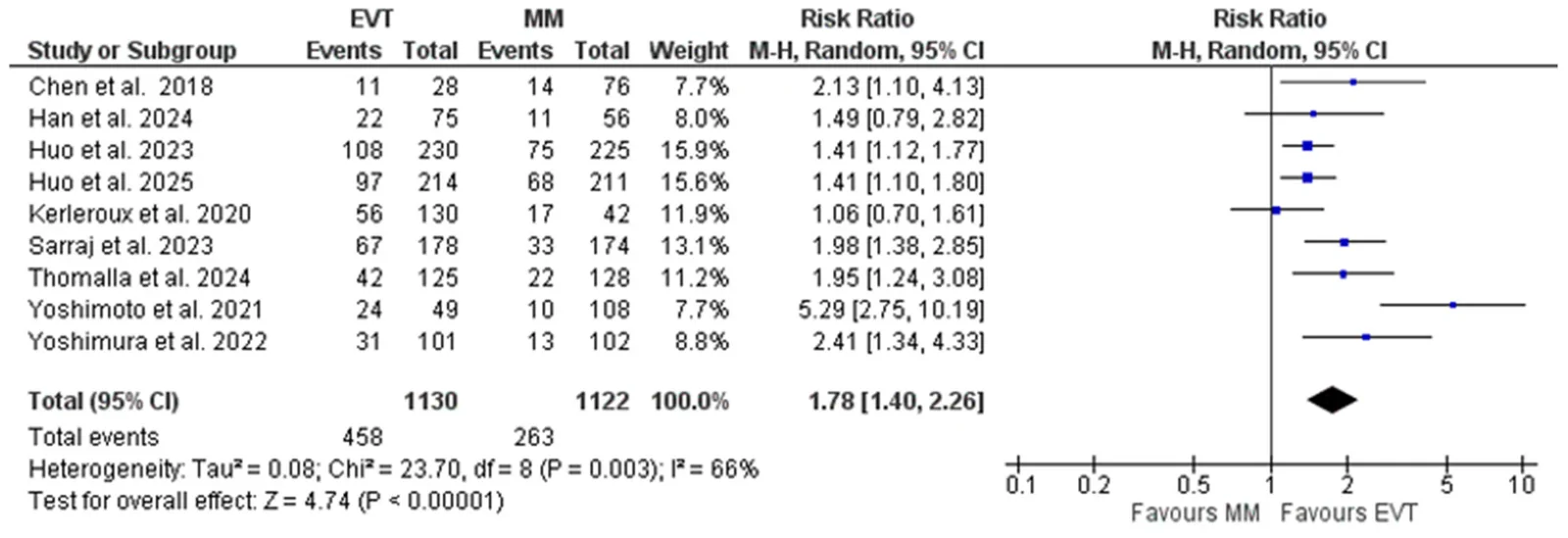
RR of mRS (0–3) [8,9,10,11,24,25,26,27,28].

Similarly, the rate of achieving an mRS score of (0–3) at 3 months was significantly higher in the EVT group compared to the MM group (Figure 5, RR = 1.78, 95% CI [1.40, 2.26], *p* < 0.00001).

**Figure 6:**
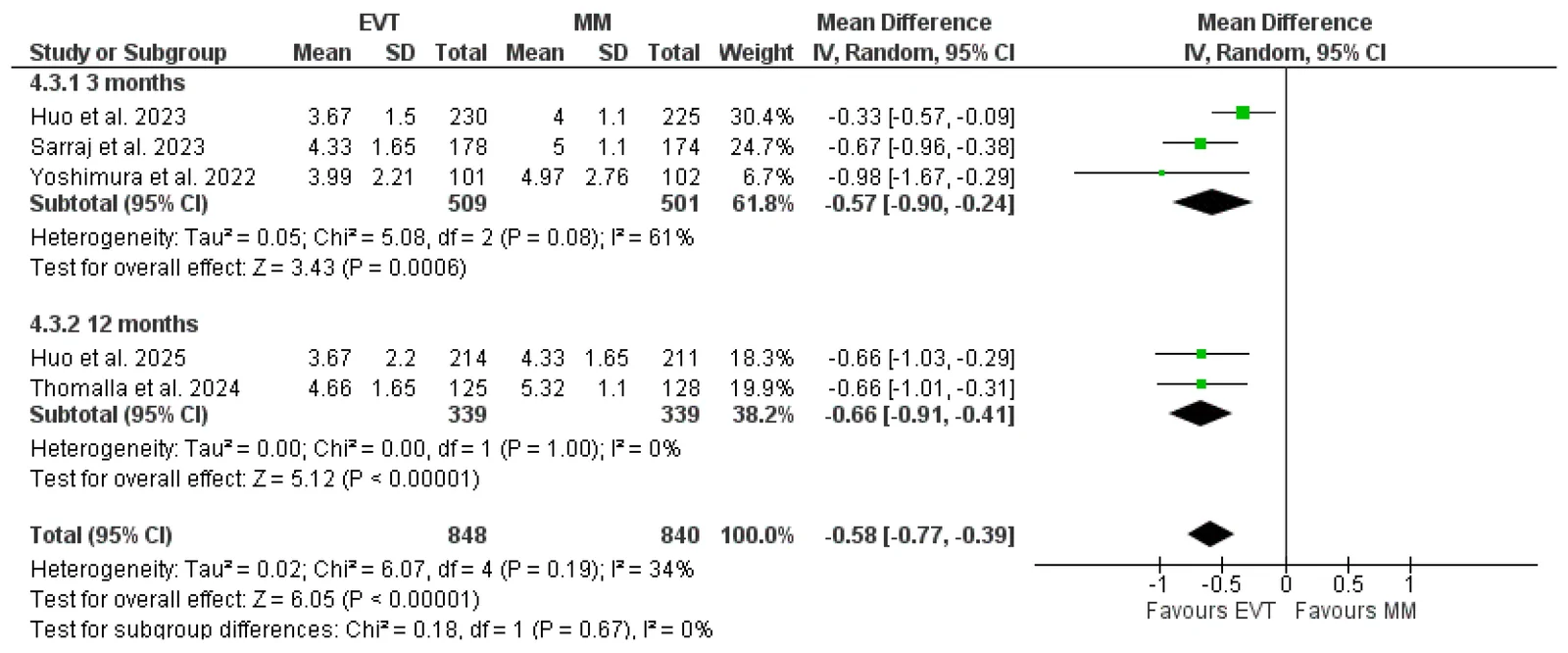
MD of mean mRS, stratified by follow-up duration (3 months vs. 12 months) [8,9,10,11,24].

Because Huo et al. [24] (2025) and Thomalla et al. [11] (2024) report the mean mRS at 12 months rather than at 3 months, Figure 6 stratifies the analysis by follow-up duration: at 3 months (Huo et al., 2023 [10], Sarraj et al., 2023 [9], and Yoshimura et al., 2022 [8]), the mean mRS was significantly lower with EVT (MD = −0.57, 95% CI [−0.90, −0.24], *p* = 0.0006, I^2^ = 61%); at 12 months (Huo et al., 2025 [24] and Thomalla et al., 2024 [11]), the mean mRS was likewise significantly lower with EVT (MD = −0.66, 95% CI [−0.91, −0.41], *p* < 0.00001, I^2^ = 0%). There was no significant subgroup difference between the two timepoints (*p* = 0.67), consistent with the subgroup analysis in Table 4 and Section 3.5.4.

#### 3.5.3. Safety Outcomes

**Figure 7:**
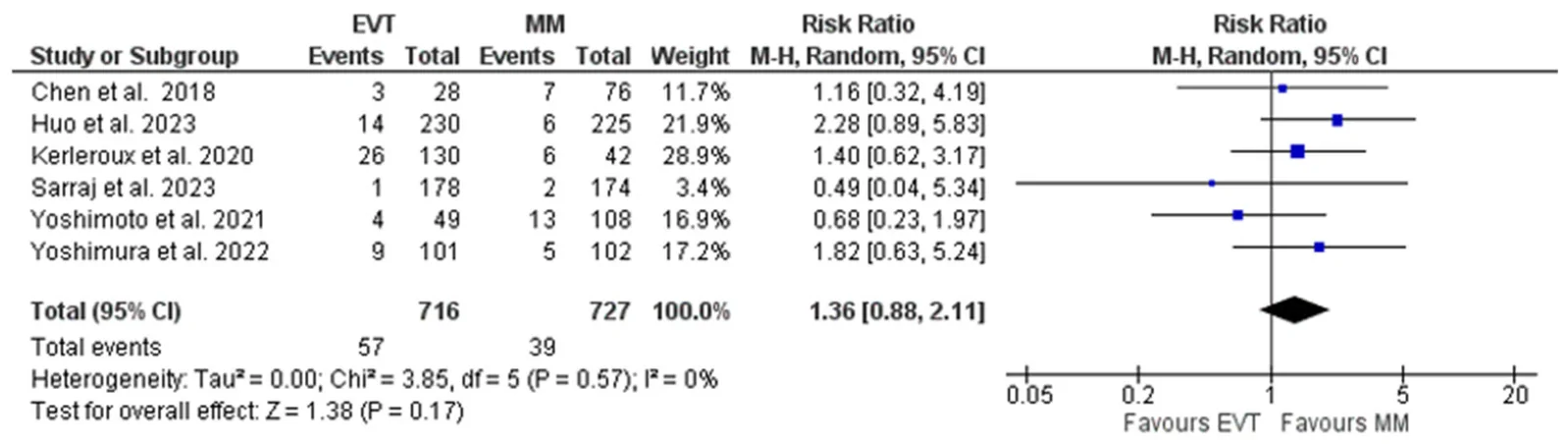
RR of sICH [8,9,10,26,27,28].

When safety profiles were compared, we found that there was no significant difference between EVT and MM in the rates of sICH at 3 months (Figure 7, RR = 1.36, 95% CI [0.88, 2.11], *p* = 0.17).

**Figure 8:**
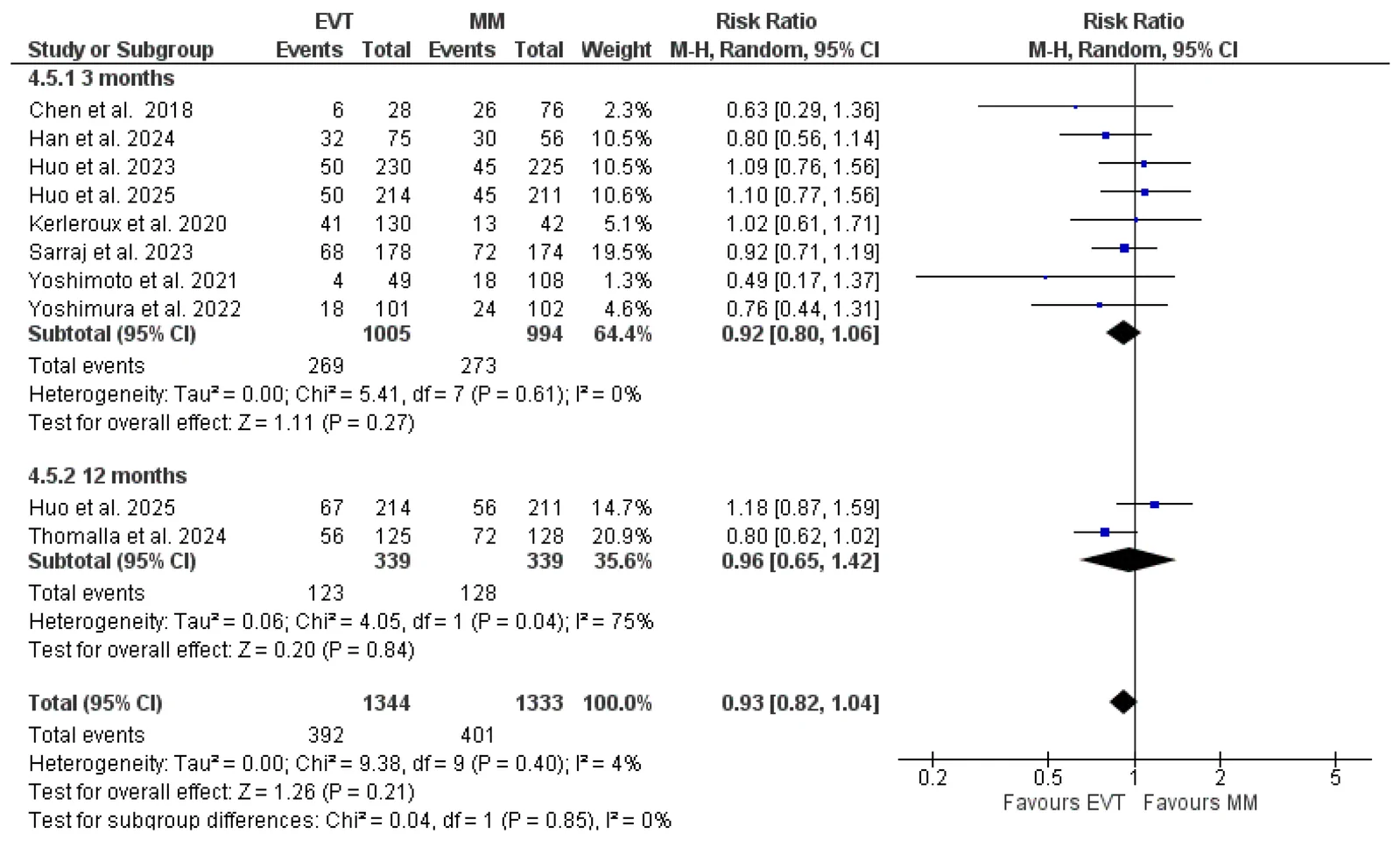
RR of mortality rate, stratified by follow-up duration (3 months vs. 12 months) [8,9,10,24,25,26,27,28].

As with the mean mRS analysis, Thomalla et al. [25] (2024) report mortality at 12 months only, so Figure 8 now stratifies mortality by follow-up duration: at 3 months (8 studies), mortality did not differ significantly between groups (RR = 0.92, 95% CI [0.80, 1.06], *p* = 0.27, I^2^ = 0%); at 12 months (Huo et al., 2025 [24] and Thomalla et al., 2024 [11]), mortality likewise did not differ significantly (RR = 0.96, 95% CI [0.65, 1.42], *p* = 0.84, I^2^ = 75%). There was no significant subgroup difference between timepoints (*p* = 0.85), consistent with Table 4.

#### 3.5.4. Subgroup Analysis

As shown in Table 4, subgroup analysis was performed between the studies based on study type, country of origin, and follow-up duration whenever feasible. The outcomes that were eligible for analysis based on study design were the rates of mRS (0–2), mRs (0–3), sICH, and mortality. For mRS (0–2) and mRS (0–3) outcomes, the RCT group showed insignificant heterogeneity between the studies compared to the cohort group (Figures S1 and S2; respectively). Conversely, no change in heterogeneity was found in the sICH rate and mortality rate (Figures S3 and S4; respectively). Despite this, our analysis revealed that there was no significant difference between the subgroups for all four outcomes (*p* = 0.85, *p* = 0.58, *p* = 0.25, *p* = 0.38; respectively). Similarly, all four outcomes were eligible for analysis based on country of origin. There were no changes in heterogeneity or effect sizes, and ultimately no significant difference between the subgroups (Figures S5–S8; *p* = 0.47, *p* = 0.51, *p* = 0.80, *p* = 0.76; respectively). Regarding follow-up duration, mean mRS score and mortality rates showed no significant difference between the subgroups for both outcomes (Figure 6 and Figure 8; *p* = 0.67, *p* = 0.85; respectively).

#### 3.5.5. Sensitivity Analysis

A leave-one-out sensitivity analysis was performed for all our outcomes to see the effect of removing studies on the overall effect estimates and heterogeneity. Kerleroux et al. (2020) [26] appeared to be the cause of heterogeneity between the studies for achieving mRS (0–2) (Figure S9). Similarly, Yoshimoto et al. (2021) [28] was found to be the cause of heterogeneity in the studies for achieving mRS (0–3) decreasing it from 66% to 33%; *p* = 0.17 (Figure S10). Lastly, removing Huo et al. (2023) [10] caused the heterogeneity between the studies to become 0% for the mean mRS outcome (Figure S11). Importantly, using the updated 2026 large-core EVT trial criteria, a comprehensive subgroup examination, together with this leave-one-out sensitivity analysis, confirmed that the overall direction and magnitude of EVT’s benefit over MM remained essentially unchanged even after individually excluding the studies most responsible for heterogeneity, indicating that the efficacy signal reported here is not driven by any single trial and represents a genuine strength of the current evidence synthesis.

#### 3.5.6. Publication Bias

**Figure 9:**
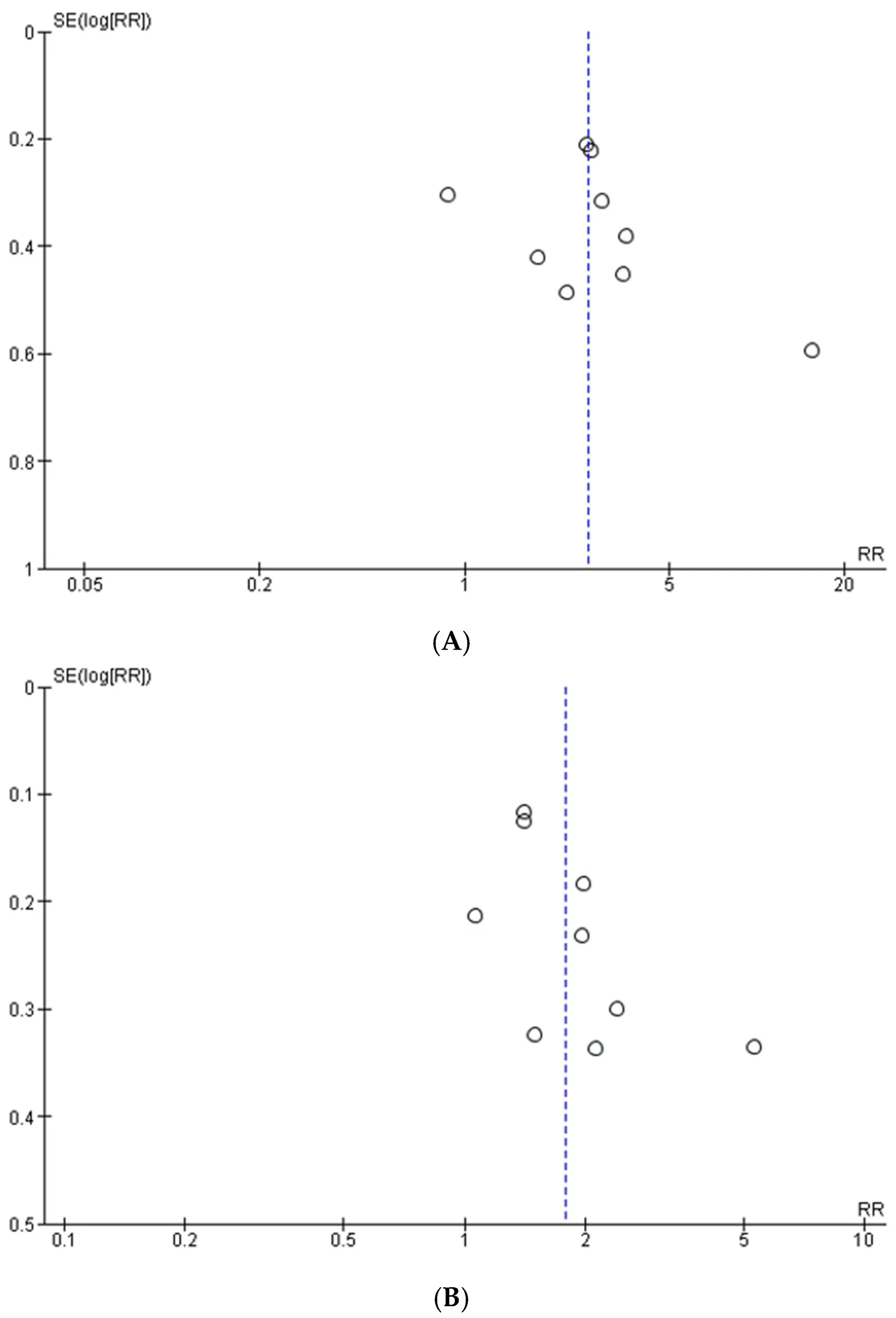
(**A**) Funnel plot of mRS (0–2). (**B**) Funnel plot of mRS (0–3).

Due to the sufficient number of studies, funnel plots were evaluated for only some outcomes, including mRS (0–2) and mRS (0–3); both of which revealed some asymmetry primarily driven by a single small study, Kerleroux et al. (2020) [26], reporting a large treatment effect on the right, alongside a relative absence of small studies with null or negative findings on the left. This reflects a possibility of publication bias, although a formal trim-and-fill adjustment was not performed given the small number of studies per outcome (fewer than 10), which limits the reliability of trim-and-fill and related regression-based methods; this asymmetry is therefore reported as a limitation of the current evidence base rather than corrected numerically (Figure 9A,B; respectively).

## 4. Discussion

This systematic review and meta-analysis synthesized data from 9 eligible studies (5 RCTs and 4 cohorts, comprising a total sample size of *n* = 2252) to evaluate the comparative efficacy and safety of EVT versus MM alone in patients presenting with AIS secondary to large vessel occlusion. Our primary efficacy findings demonstrate a robust clinical benefit for the interventional; specifically, patients undergoing EVT were 2.64 times more likely to achieve an excellent functional outcome (mRS 0–2) and 1.78 times more likely to achieve functional independence (mRS 0–3) compared to those receiving MM alone. In contrast to these efficacy outcomes, safety profiles did not differ significantly between the two treatment groups, a finding that remained robust across all subgroup analyses. Although the rate of sICH was marginally higher in the EVT group (RR = 1.36), this trend did not achieve statistical significance (*p* = 0.17). Conversely, a slight trend toward reduced mortality was observed in the EVT group (RR = 0.89), though this likewise fell short of statistical significance (*p* = 0.06). Notably, both safety endpoints exhibited zero statistical heterogeneity.

The pooled analysis revealed that patients undergoing EVT were significantly more likely to achieve an excellent functional outcome (mRS 0–2) (RR = 2.64, *p* < 0.00001) and a higher rate of functional independence (mRS 0–3) (RR = 1.78, *p* < 0.00001) compared to those receiving MM alone. Notably, a high degree of statistical heterogeneity was observed across both overall efficacy endpoints. Crucially, this advantage was not an artifact of study design, as RCTs of mRS 0–2 (RR = 2.68, *p* < 0.00001) and cohort studies (RR = 3.01, *p* < 0.00001) showed similar results. Notably, the overall heterogeneity was primarily driven by the cohort studies (I^2^ = 86%). Similarly, the benefit of EVT remained significant across both RCTs (RR = 1.64, *p* < 0.00001) and cohort studies (RR = 2.01, *p* = 0.05) of mRS 0–3. Similarly, the overall heterogeneity was primarily driven by the cohort studies (I^2^ = 83%). These findings align closely with a prior meta-analysis by Li et al. [29], which demonstrated that among stroke patients with a large ischemic core, EVT markedly improved the likelihood of achieving mRS 0–2 in both RCTs (RR = 2.59, *p* < 0.001, I^2^ = 0%) and cohort studies (RR = 3.39, *p* < 0.001, I^2^ = 74%). Li et al. reported comparable trends for the mRS 0–3 endpoint across RCTs (RR = 1.78, *p* < 0.001, I^2^ = 58%) and cohort studies (RR = 2.33, *p* < 0.001, I^2^ = 78%). Conversely, our robust efficacy data stand in contrast to the meta-analysis and trial sequential analysis by Chen et al. [16], which emphasized that EVT did not yield a statistically significant impact on excellent outcomes (*p* = 0.06) or favorable functional independence outcomes (*p* = 0.04). This discrepancy could be attributed to variations in sample size configurations, stricter patient selection criteria regarding baseline core volumes, or differences in the inclusion ratios of prospective randomized trials versus real-world observational cohorts. On the trial level, RCTs further validate the therapeutic advantage of mechanical intervention. Specifically, the Chinese trial demonstrated that the rate of achieving an mRS 0–2 score was 30.0% in the EVT group compared to 11.6% in the MM group, while the proportion of patients achieving an mRS 0–3 was 47.0% and 33.3%, respectively [10]. Similarly, the SELECT2 trial corroborated these efficacy trends, reporting significantly higher rates of mRS 0–2 outcomes (20.3% vs. 7.0%) and functional independence defined by mRS 0–3 (37.9% vs. 18.7%) in the EVT group relative to MM alone [9]. Collectively, these trials underscore the robust clinical efficacy of EVT in patients presenting with AIS and large infarct cores, strongly mirroring the conclusions of our pooled randomized analysis.

Interestingly, geographic subgroups revealed distinct variations in statistical significance. Regarding the mRS 0–2 endpoint, EVT was highly effective within the Asian populations (RR = 2.98, *p* < 0.00001). Conversely, within European populations, the treatment effect directionally favored EVT (RR = 2.07) but failed to achieve statistical significance (*p* = 0.11). This lack of significance is highly likely an artifact of an underpowered sample size characterized by fewer overall patients. The functional independence mRS 0–3 showed that mechanical intervention yielded significantly superior outcomes in Asia (RR = 1.92, *p* = 0.0001), and, unlike the mRS 0–2 endpoint, this benefit reached statistical significance in the European subgroup as well (RR = 1.61, *p* = 0.02). The non-significant European result for mRS 0–2 (*p* = 0.11) most likely reflects an underpowered sample size rather than a true absence of effect, and larger multicenter cohorts are needed to confirm the magnitude of benefit in this subgroup.

The ANGEL-ASPECT trial demonstrated that patients who underwent EVT for large infarct cores sustained favorable clinical outcomes over time, with mRS 0–2 rates remaining stable between 3 months and 12 months (29.4% vs. 30.4%, respectively). Conversely, a delayed gain in functional independence was observed within the MM group from 90 days to 1 year (10.9% vs. 17.1%). Regarding independent ambulation mRS 0–3, event rates remained relatively stable between 90 days and 1 year across both the EVT (45.3% vs. 50.0%) and MM (32.2% vs. 35.6%) cohorts [24]. Our subgroup analysis confirmed that there was no statistically significant difference between the overall treatment effects observed at 3 months versus 12 months (−0.57 vs. −0.66, respectively).

Regarding safety outcomes, the rate of sICH was slightly higher in the EVT group than in the MM group (RR = 1.36); however, this difference was not statistically significant (*p* = 0.17), and no heterogeneity was observed (I^2^ = 0%). This lack of significance persisted across both study designs in the subgroup analyses, including both the RCTs alone (RR = 1.84, *p* = 0.08, I^2^ = 0%) and the cohort studies alone (RR = 1.09, *p* = 0.77, I^2^ = 0%). These findings align closely with a prior meta-analysis by Li et al. [29], which similarly reported no statistically significant difference in sICH rates between the EVT and MM groups across both RCTs (*p* = 0.07) and cohort studies (*p* = 0.95). Furthermore, a meta-analysis by Chen et al. demonstrated a comparable safety profile, finding no significant difference in sICH risk between the two groups (*p* = 0.41) [16]. At the trial level, three high-quality randomized controlled trials—RESCUE-Japan LIMIT [8], ANGEL-ASPECT [10], and SELECT2 [9]—similarly demonstrated no significant differences in sICH rates between the EVT and MM groups. Taken together, these findings indicate that EVT does not conclusively increase the risk of sICH compared to MM alone. Furthermore, subgroup analysis by geographical region revealed no significant differences in safety outcomes. The risk of sICH was comparable between the EVT and MM cohorts in both the Asian (RR = 1.41, *p* = 0.21) and European (RR = 1.25, *p* = 0.57) subgroups.

Mortality rates were statistically comparable between the EVT and MM groups in both the RCTs (RR = 0.92, *p* = 0.22, I^2^ = 0%) and the cohort studies (RR = 0.80, *p* = 0.10, I^2^ = 0%). While the meta-analysis by Li et al. demonstrated similar non-significant results among RCTs (RR = 0.95, *p* = 0.61, I^2^ = 0%), their analysis of cohort studies conversely showed that EVT significantly reduced mortality in patients with a large ischemic core (RR = 0.60, *p* < 0.001, I^2^ = 49%) [29]. This discrepancy between the cohort findings likely stems from the inclusion of less observational data in our study, as well as the inherent treatment selection biases in real-world cohort registries. Furthermore, our subgroup analyses revealed no significant differences in mortality when stratified by geographical region (Asian vs. European populations) or by follow-up duration (3 vs. 12 months).

Overall, the demonstrated efficacy of EVT in this study supports its clinical benefit in patients with large core ischemic strokes. Notably, the lack of heterogeneity (I^2^ = 0%) among the included RCTs strengthens the evidence favoring EVT for achieving superior long-term functional outcomes, though further long-term validation remains warranted. Furthermore, the acceptable safety profile of EVT relative to MM supports its broader integration into clinical practice. However, to optimize patient outcomes and minimize procedural risks, the implementation of EVT should be restricted to specialized centers and performed by experienced neurointerventionalists, such as stroke neurologists or neurosurgeons with specific endovascular expertise.

Our study has several limitations that warrant consideration. First, although randomized and non-randomized studies were analyzed separately in all subgroup analyses, the overall pooled effect estimates combine RCTs and cohort studies into a single summary measure. Because cohort studies are more susceptible to selection and confounding bias than RCTs, the overall pooled estimates should be interpreted with caution, and the RCT-only subgroup results should be regarded as the more robust reflection of EVT’s true efficacy. Second, a variation of neuroimaging modalities was used across the included studies to assess ischemic changes. Third, the criteria used to define a “large ischemic core” varied among the trials, which may introduce selection variability. Fourth, we observed high heterogeneity among the cohort studies regarding efficacy outcomes, which necessitates caution when pooling observational data. Consequently, further standardized analyses and high-quality trials are needed to account for these variables and definitively confirm the overall benefit of EVT compared to MM alone.

## 5. Conclusions

In conclusion, this systematic review and meta-analysis provides contemporary evidence regarding the clinical utility of thrombectomy. Our findings demonstrate that in patients presenting with large-core ischemic strokes secondary to LVO, EVT significantly improves functional outcomes compared to MM alone, while maintaining an acceptable safety profile. Although these data strongly support the use of EVT in this population, the results of ongoing and future randomized controlled trials will be essential to further validate and refine these findings.

## Figures and Tables

**Table 1:** A summary of the characteristics of the included studies.

Study ID	Country	Design	Follow-Up	Number of Participants			Mean Age of Participants, Years (±SD)		Sex, *n* (%); M/F			
									Female		Male	
				EVT	MM	Total	EVT	MM	EVT	MM	EVT	MM
Huo et al., 2025 [24]	China	RCT	3 months & 12 months	214	211	425	67.33 ± 8.89	66.33 ± 10.37	88 (41.1%)	74 (35.0%)	126 (58.9%)	137 (65.0%)
Yoshimura et al., 2022 [8]	Japan	RCT	3 months	101	102	203	76.60 ± 10.00	75.70 ± 10.20	46 (45.5%)	44 (43.1%)	55 (54.5%)	58 (56.9%)
Thomalla et al., 2024 [11]	Europe, Canada	RCT	12 months	125	128	253	73.00 ± 11.85	72.67 ± 11.85	56 (45%)	67 (52%)	69 (55%)	61 (48%)
Huo et al., 2023 [10]	China	RCT	3 months	230	225	455	67.33 ± 8.89	66.33 ± 10.37	95 (41.3%)	81 (36.0%)	135 (58.7%)	144 (64.0%)
Sarraj et al., 2023 [9]	Multinational	RCT	3 months	178	174	352	66.33 ± 12.59	66.67 ± 12.59	71 (39.9%)	74 (42.5%)	107 (60.1%)	100 (57.5%)
Han et al., 2024 [25]	China	Cohort study	3 months	75	56	131	67.20 ± 14.20	73.50 ± 12.40	33 (44.0%)	25 (44.6%)	42 (56.0%)	31 (55.4%)
Kerleroux et al., 2020 [26]	France, Switzerland	Cohort study	3 months	130	42	172	66.20 ± 15.00	77.70 ± 13.50	53 (40.8%)	9 (21.4%)	77 (59.23%)	33 (78.5%)
Chen et al., 2018 [27]	China	Cohort study	3 months	28	76	104	68.40 ± 14.00	73.80 ± 11.80	6 (21.4%)	26 (34.2%)	22 (78.6%)	50 (65.8%)
Yoshimoto et al., 2021 [28]	Japan	Cohort study	3 months	49	108	157	77.33 ± 10.37	77.67 ± 14.81	N/A	N/A	N/A	N/A
Total: 9				Total: 1130	Total: 1122	Total: 2252	Total: 69.64 ± 12.21		Total: 848 (40.5%)		Total: 1247 (59.5%)	

Studies are grouped by design (RCTs listed first, followed by cohort studies) and ordered chronologically by publication year within each group; forest plots (Figures 4–8) list studies alphabetically by first author, which is the RevMan 5 default sorting order. Huo et al. appears twice because it refers to two distinct publications by the same lead author (the 2023 [10] ANGEL-ASPECT trial report and the 2025 [24] report of its 1-year outcomes). Abbreviations: EVT, Endovascular Therapy; MM, Medical Management; SD, Standard Deviation; RCT, Randomized Controlled Trial.

**Table 2:** GRADE assessment of outcomes.

Outcomes	Anticipated Absolute Effects * (95% CI)		Relative Effect (95% CI)	№ of Participants (Studies)	Certainty of the Evidence (GRADE)
	Risk with MM	Risk with EVT			
mRS (0–2) follow-up: 3 months	92 per 1000	242 per 1000 (164 to 358)	RR 2.64 (1.79 to 3.90)	2252 (5 RCTs 4 Cohorts)	⨁⨁⨁○ Moderate
mRS (0–3) follow-up: 3 months	234 per 1000	417 per 1000 (328 to 530)	RR 1.78 (1.40 to 2.26)	2252 (5 RCTs 4 Cohorts)	⨁⨁⨁○ Moderate
Mean mRS follow-up: 3 months		MD 0.57 lower (0.90 lower to 0.24 lower)	-	1010 (3 RCTs)	⨁⨁⨁○ Moderate
Mean mRS follow-up: 12 months		MD 0.66 lower (0.91 lower to 0.41 lower)	-	678 (2 RCTs)	⨁⨁⨁⨁ High
sICH	54 per 1000	73 per 1000 (47 to 113)	RR 1.36 (0.88 to 2.11)	1443 (3 RCTs 3 Cohorts)	⨁⨁⨁⨁ High
Mortality rate follow-up: 3 months	275 per 1000	253 per 1000 (220 to 292)	RR 0.92 (0.80 to 1.06)	1999 (4 RCTs 4 Cohorts)	⨁⨁⨁⨁ High
Mortality rate follow-up: 12 months	378 per 1000	363 per 1000 (246 to 537)	RR 0.96 (0.65 to 1.42)	678 (2 RCTs)	⨁⨁⨁○ Moderate

* The risk in the intervention group (and its 95% confidence interval) is based on the assumed risk in the comparison group and the relative effect of the intervention (and its 95% CI). CI: confidence interval; MD: mean difference; RR: risk ratio. Certainty for mRS (0–2) and mRS (0–3) was downgraded one level for inconsistency (moderate-to-substantial statistical heterogeneity, I^2^ = 67% and 66% overall, rising to 86% and 83% in the cohort-study subgroup) and the “some concerns” RoB 2 rating of several contributing RCTs; sICH and mean mRS/mortality at 12 months showed low heterogeneity and were not downgraded. Certainty for mean mRS at 3 months was downgraded one level for inconsistency (I^2^ = 61%, driven by the RCT subgroup at this timepoint). Certainty for mortality at 12 months was downgraded one level for inconsistency (I^2^ = 75%) and imprecision (wide 95% CI crossing unity, based on only 2 studies). GRADE Working Group grades of evidence: High certainty: we are very confident that the true effect lies close to that of the estimate of the effect. Moderate certainty: we are moderately confident in the effect estimate; the true effect is likely to be close to the estimate of the effect, but there is a possibility that it is substantially different. Low certainty: our confidence in the effect estimate is limited; the true effect may be substantially different from the estimate of the effect. Very low certainty: we have very little confidence in the effect estimate; the true effect is likely to be substantially different from the estimate of effect.

**Table 3:** Summary of outcomes.

Outcomes		Number of Studies	Number of Patients (EVT/MM)	Heterogeneity		Model Used (Fixed/Random)	Effect Size		
				*p*	I^2^ (%)		MD/RR	95% CI	*p*
Efficacy	mRS at 3 months (0–2)	9	1130/1122	0.002	67	Random	2.64	[1.79, 3.90]	<0.00001 *
	mRS at 3 months (0–3)	9	1130/1122	0.003	66	Random	1.78	[1.40, 2.26]	<0.00001 *
	Mean mRS at 3 months	5	848/840	0.19	34	Random	−0.58	[−0.77, −0.39]	<0.00001 *
	Mean mRS at 12 months	2	339/339	1.00	0	Random	−0.66	[−0.91, −0.41]	<0.00001 *
Safety	Symptomatic intracranial hemorrhage	6	716/727	0.57	0	Random	1.36	[0.88, 2.11]	0.17
	Mortality rate at 3 months	9	1130/1122	0.60	0	Random	0.89	[0.78, 1.01]	0.06
	Mortality rate at 12 months	2	339/339	0.04	75	Random	0.96	[0.65, 1.42]	0.84

* *p*-value is significant. Abbreviations: EVT, Endovascular Therapy; MM, Medical Management; MD, Mean Difference; RR, Risk Ratio; CI, Confidence Interval; mRS, Modified Rankin Scale.

**Table 4:** Summary of subgroup analysis.

Subgroup Analysis Based on Study Design							
Outcomes	Effect Size			Heterogeneity		*p*-Value for Subgroup Difference	Number of Studies
	MD/RR	95% CI	*p*	*p*	I^2^ (%)		
mRS at 3 months (0–2)							
RCT	2.68	[2.10, 3.42]	<0.00001 *	0.80	0	0.85	5
Cohort	3.01	[0.94, 9.71]	0.06	<0.0001	86		4
mRS at 3 months (0–3)							
RCT	1.64	[1.36, 1.98]	<0.00001 *	0.18	37	0.58	5
Cohort	2.01	[1.00, 4.03]	0.05 *	0.0007	83		4
Symptomatic intracranial hemorrhage							
RCT	1.84	[0.94, 3/61]	0.08	0.50	0	0.25	3
Cohort	1.09	[0.61, 1.94]	0.77	0.57	0		3
Mortality rate (follow-up: 3 months)							
RCT	0.92	[0.79, 1.05]	0.22	0.46	0	0.38	5
Cohort	0.80	[0.61, 1.04]	0.10	0.54	0		4
**Subgroup Analysis Based on Country**							
**Outcomes**	**Effect Size**			**Heterogeneity**		***p*** **-Value for Subgroup Difference**	**Number of Studies**
	**MD/RR**	**95% CI**	***p***	***p***	**I^2^ (%)**		
mRS at 3 months (0–2)							
Asia	2.98	[1.99, 4.46]	<0.00001 *	0.07	51	0.47	6
Europe	2.07	[0.84, 5.06]	0.11	0.003	82		3
mRS at 3 months (0–3)							
Asia	1.92	[1.38, 2.67]	<0.0001 *	0.00	72	0.51	6
Europe	1.61	[1.07, 2.41]	0.02 *	0.05	66		3
Symptomatic intracranial hemorrhage							
Asia	1.41	[0.82, 2.44]	0.21	0.37	4	0.80	4
Europe	1.25	[0.58, 2.72]	0.57	0.41	0		2
Mortality rate (follow-up: 3 months)							
Asia	0.91	[0.75, 1.10]	0.31	0.39	5	0.76	6
Europe	0.87	[0.74, 1.03]	0.11	0.59	0		3
**Subgroup Analysis Based on Follow-Up Duration**							
**Outcomes**	**Effect Size**			**Heterogeneity**		***p*** **-Value for Subgroup Difference**	**Number of Studies**
	**MD/RR**	**95% CI**	***p***	***p***	**I^2^ (%)**		
Mean mRS							
3 months	−0.57	[−0.90, −0.24]	0.0006 *	0.08	61	0.67	3
12 months	−0.66	[−0.91, −0.41]	<0.00001 *	1.00	0		2
Mortality rate							
3 months	0.92	[0.80, 1.06]	0.27	0.61	0	0.85	8
12 months	0.96	[0.65, 1.42]	0.84	0.04 *	75		2

* *p*-value is significant. Abbreviations; MD, Mean Difference; RR, Risk Ratio; CI, Confidence Interval; mRS, Modified Rankin Scale.

## Data Availability

The corresponding author can provide the supporting data for this study upon reasonable request.

## Supplementary Materials

The following supporting information can be downloaded at https://www.mdpi.com/article/10.3390/neurosci7050102/s1, Table S1: Baseline Characteristics [8,9,10,11,24,25,26,27,28]; Figure S1: mRS at 3 Months (0–2), RCT vs. Cohort [8,9,10,11,24,25,26,27,28]; Figure S2: mRS at 3 Months (0–3), RCT vs. Cohort [8,9,10,11,24,25,26,27,28]; Figure S3: sICH Rate, RCT vs. Cohort [8,9,10,26,27,28]; Figure S4: Mortality Rate, RCT vs. Cohort [8,9,10,11,24,25,26,27,28]; Figure S5: mRS at 3 Months (0–2), Asia vs. Europe [8,9,10,11,24,25,26,27,28]; Figure S6: mRS at 3 Months (0–3), Asia vs. Europe [8,9,10,11,24,25,26,27,28]; Figure S7: sICH Rate, Asia vs. Europe [8,9,10,26,27,28]; Figure S8: Mortality Rate, Asia vs. Europe [8,9,10,11,24,25,26,27,28]; Figure S9: Sensitivity Analysis for mRS at 3 Months (0–2) Excluding Kerleroux et al., 2020 [8,9,10,11,24,25,26,27,28]; Figure S10: Sensitivity Analysis for mRS at 3 Months (0–3) Excluding Yoshimoto et al., 2021 [8,9,10,11,24,25,26,27,28]; and Figure S11: Sensitivity Analysis for Mean mRS Excluding Huo et al., 2023 [8,9,10,11,24].
