# Supplementary material for: Endovascular Thrombectomy for Acute Ischemic Stroke Due to Large Vessel Occlusion with Large Ischemic Core: A Systematic Review and Meta-Analysis
Source: NeuroSci. 2026 Sep 17;7(5):102. doi: 10.3390/neurosci7050102 (PMC13600027; doi:10.3390/neurosci7050102)

Table S1. Baseline Characteristics [8–11,24–28].

Study ID	Median NIHSS score at baseline (IQR)		Occlusion site — no. (%)								ASPECTS value (Median value (IQR))		Median infarct-core volume (IQR) — ml		Intravenous thrombolysis — no. (%)		Interval between the time of stroke onset and time of randomization (Median (IQR), min)		Imaging method used for enrolment				Affected hemisphere				Wake-up stroke, n (%)	
	EVT	MM	Internal carotid artery		M1 segment		M2 segment		Tandem occlusions		EVT	MM	EVT	MM	EVT	MM	EVT	MM	CT		MRI		Right		Left		EVT	MM
			EVT	MM	EVT	MM	EVT	MM	EVT	MM									EVT	MM	EVT	MM	EVT	MM	EVT	MM		
Huo et al., 2025	16 (13–20)	15 (12–19)	79 (36.9%)	77 (36.5%)	133 (62.2%)	133 (63.0%)	2 (0.9%)	1 (0.5%)	39 (18.2%)	32 (15.2%)	3 (3–4)	3 (3–4)	60 (28–86)	64 (34–88)	51 (23.8%)	52 (24.6%)	459 (299–713)	456 (305–781)	All patients		38 patients overall had MRI ADC-based infarct assessment		113 (52.8%)	116 (55.0%)	101 (47.2%)	95 (45.0%)	64 (30.0%)	74 (35.0%)
Yoshimura et al., 2022	22 (18–26)	22 (17–26)	47 (46.5%)	49 (48.0%)	74 (73.3%)	70 (68.6%)	0	3 (2.9%)	20 (19.8%)	20 (19.6%)	3 (3–4)	4 (3–4)	94 (66–152)	110 (74–140)	27 (26.7%)	29 (28.4%)	229 (144–459)	214 (142–378)	13	15	88	87	N/A	N/A	N/A	N/A	N/A	N/A
Thomalla et al., 2024	19 (16–22)	18 (15–22)	41 (33%)	37 (29%)	83 (66%)	88 (69%)	0 (0%)	1 (1%)	8 (6%)	7 (5%)	N/A	N/A	N/A	N/A	49 (39%)	44 (34%)	(72–210)	60 (72–216)	104 (83%)	104 (81%)	21 (17%)	24 (19%)	N/A	N/A	N/A	N/A	N/A	N/A
Huo et al., 2023	16 (13–20)	15 (12–19)	83 (36.1%)	81 (36.0%)	145 (63.0%)	142 (63.1%)	2 (0.9%)	2 (0.9%)	41 (17.8%)	35 (15.6%)	3 (3–4)	3 (3–4)	60.5 (29–86)	63 (31–86)	66 (28.7%)	63 (28.0%)	453 (299–712)	463 (305–781)	Majority		38 patients total for infarct-core assessment		N/A	N/A	N/A	N/A	69 (30.0%)	78 (34.7%)
Sarraj et al., 2023	19 (15–23)	19 (15–22)	80 (44.9%)	66 (37.9%)	91 (51.1%)	100 (57.5%)	7 (3.9%)	8 (4.6%)	56 (31.5%)	44 (25.3%)	4 (3–5)	4 (4–5)	74 (50–111.5)	77 (50.3–105)	37 (20.8%)	30 (17.3%)	544.2	587.4	98.3%	97.1%	1.7%	2.9%	55.1%	56.3%	156		N/A	N/A
Han et al., 2024	18 (14–21)	17 (15–24)	52.0%	48.2%	36.0%	41.1%	12.0%	10.7%	2.7%	N/A	N/A	N/A	121.6 (95.7–164.9)	148.5 (101.5–224.2)	33.3%	14.3%	305 (166–488)	458 (268–709)	N/A	N/A	N/A	N/A	48%	42.9%	52%	57.1%	21.3%	32.1%
Kerleroux et al., 2020	18.7±4.2	18±5.1	24 (18.5%)	7 (16.7%)	N/A	N/A	N/A	N/A	N/A	N/A	DWI-ASPECTS inclusion range 0–6		104 ± 37.7	96.7 ± 33	63 (48.5%)	42 (100%)	164 ± 135.2	152.7 ± 108.5	N/A	N/A	N/A	N/A	71 (54.6%)	26 (61.9%)	59 (45.4%)	16 (38.1%)	N/A	N/A

**Chen et al., 2018**	**16 (12–18)**	**16 (13–19)**	**14 (50.0%)**	**31 (40.8%)**	**N/A**	**N/A**	**N/A**	**N/A**	**N/A**	**N/A**	**N/A**	**N/A**	**97.8 (80.8–115.7)**	**114.6 (85.4–143.9)**	**N/A**	**N/A**	**156 (95–224)**	**185 (112–231)**	**CT = 91 (87.5%)<br>MRI = 13 (12.5%)**				**N/A**	**N/A**	**N/A**	**N/A**	**N/A**	**N/A**
**Yoshimoto et al., 2021**	**21 (18–24)**	**20 (16–26)**	**20 (40.8)**	**43 (54.5)**	**16 (32.7)**	**35 (32.4)**	**3 (6.1)**	**6 (5.6)**	**9 (18.4)**	**3 (2.8)**	**5 (4–6)<br>6 (4–6)<br>3 (3–6)<br>4 (3–5)<br>3 (1–5)<br>2 (1–3)**		**80 (74–87)<br>107 (101–117)<br>178 (175–194)<br>85 (77–91)<br>113 (107–124)<br>182 (150–210)**		**19 (38.8)**	**3 (2.8)**	**N/A**	**N/A**	**N/A**	**N/A**	**N/A**	**N/A**	**N/A**	**N/A**	**N/A**	**N/A**	**N/A**	**N/A**

Abbreviations: EVT, Endovascular Therapy; MM, Medical Management; IQR, Interquartile Range; NIHSS, National Institutes of Health Stroke Scale; ASPECTS, Alberta Stroke Program Early CT Score
Studies are listed in the same order as Table 1 of the main manuscript (RCTs first, followed by cohort studies, chronologically within each group).

Figure S1. mRS at 3 Months (0–2), RCT vs. Cohort [8–11,24–28].

Study or Subgroup	EVT Events	EVT Total	MM Events	MM Total	Weight	Risk Ratio M-H, Random, 95% CI
**2.1.1 RCT**						
Huo et al. 2023	69	230	26	225	14.9%	2.60 [1.72, 3.92]
Huo et al. 2025	63	214	23	211	14.6%	2.70 [1.74, 4.19]
Sarraj et al. 2023	36	178	12	174	12.3%	2.93 [1.58, 5.45]
Thomalla et al. 2024	28	125	8	128	10.8%	3.58 [1.70, 7.56]
Yoshimura et al. 2022	14	101	8	102	9.9%	1.77 [0.78, 4.03]
**Subtotal (95% CI)**		**848**		**840**	**62.6%**	**2.68 [2.10, 3.42]**
Total events	210		77			
Heterogeneity: Tau² = 0.00; Chi² = 1.67, df = 4 (P = 0.80); I² = 0%						
Test for overall effect: Z = 7.96 (P < 0.00001)						
**2.1.2 Cohort**						
Chen et al. 2018	9	28	7	76	9.3%	3.49 [1.44, 8.48]
Han et al. 2024	15	75	5	56	8.6%	2.24 [0.87, 5.80]
Kerleroux et al. 2020	30	130	11	42	12.6%	0.88 [0.49, 1.60]
Yoshimoto et al. 2021	21	49	3	108	6.9%	15.43 [4.83, 49.30]
**Subtotal (95% CI)**		**282**		**282**	**37.4%**	**3.01 [0.94, 9.71]**
Total events	75		26			
Heterogeneity: Tau² = 1.21; Chi² = 21.68, df = 3 (P < 0.0001); I² = 86%						
Test for overall effect: Z = 1.85 (P = 0.06)						
**Total (95% CI)**		**1130**		**1122**	**100.0%**	**2.64 [1.79, 3.90]**
Total events	285		103			
Heterogeneity: Tau² = 0.22; Chi² = 24.03, df = 8 (P = 0.002); I² = 67%						
Test for overall effect: Z = 4.92 (P < 0.00001)						
Test for subgroup differences: Chi² = 0.04, df = 1 (P = 0.85), I² = 0%						

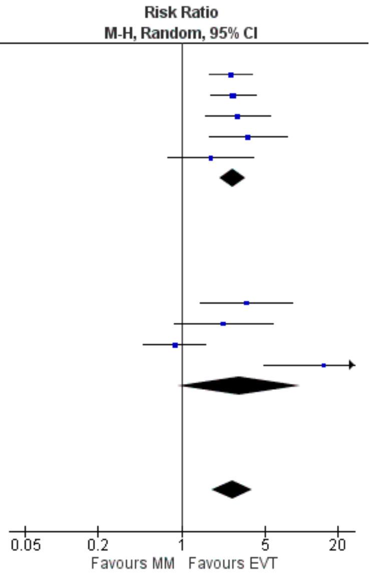

Figure S2. mRS at 3 Months (0–3), RCT vs. Cohort [8–11,24–28].

Study or Subgroup	EVT Events	EVT Total	MM Events	MM Total	Weight	Risk Ratio M-H, Random, 95% CI
**2.2.1 RCT**						
Huo et al. 2023	108	230	75	225	15.9%	1.41 [1.12, 1.77]
Huo et al. 2025	97	214	68	211	15.6%	1.41 [1.10, 1.80]
Sarraj et al. 2023	67	178	33	174	13.1%	1.98 [1.38, 2.85]
Thomalla et al. 2024	42	125	22	128	11.2%	1.95 [1.24, 3.08]
Yoshimura et al. 2022	31	101	13	102	8.8%	2.41 [1.34, 4.33]
**Subtotal (95% CI)**		**848**		**840**	**64.6%**	**1.64 [1.36, 1.98]**
Total events	345		211			
Heterogeneity: Tau² = 0.02; Chi² = 6.30, df = 4 (P = 0.18); I² = 37%						
Test for overall effect: Z = 5.16 (P < 0.00001)						
**2.2.2 Cohort**						
Chen et al. 2018	11	28	14	76	7.7%	2.13 [1.10, 4.13]
Han et al. 2024	22	75	11	56	8.0%	1.49 [0.79, 2.82]
Kerleroux et al. 2020	56	130	17	42	11.9%	1.06 [0.70, 1.61]
Yoshimoto et al. 2021	24	49	10	108	7.7%	5.29 [2.75, 10.19]
**Subtotal (95% CI)**		**282**		**282**	**35.4%**	**2.01 [1.00, 4.03]**
Total events	113		52			
Heterogeneity: Tau² = 0.41; Chi² = 17.16, df = 3 (P = 0.0007); I² = 83%						
Test for overall effect: Z = 1.97 (P = 0.05)						
**Total (95% CI)**		**1130**		**1122**	**100.0%**	**1.78 [1.40, 2.26]**
Total events	458		263			
Heterogeneity: Tau² = 0.08; Chi² = 23.70, df = 8 (P = 0.003); I² = 66%						
Test for overall effect: Z = 4.74 (P < 0.00001)						
Test for subgroup differences: Chi² = 0.30, df = 1 (P = 0.58), I² = 0%						

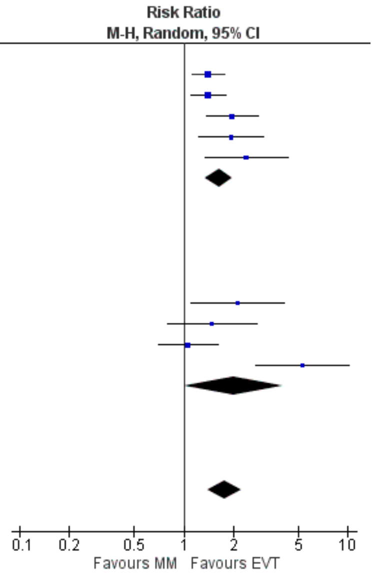

Figure S3. sICH Rate, RCT vs. Cohort [8–10,26–28].

Study or Subgroup	EVT Events	EVT Total	MM Events	MM Total	Weight	Risk Ratio M-H, Random, 95% CI
**2.4.1 RCT**						
Huo et al. 2023	14	230	6	225	21.9%	2.28 [0.89, 5.83]
Sarraj et al. 2023	1	178	2	174	3.4%	0.49 [0.04, 5.34]
Yoshimura et al. 2022	9	101	5	102	17.2%	1.82 [0.63, 5.24]
**Subtotal (95% CI)**		**509**		**501**	**42.5%**	**1.84 [0.94, 3.61]**
Total events	24		13			
Heterogeneity: Tau² = 0.00; Chi² = 1.38, df = 2 (P = 0.50); I² = 0%						
Test for overall effect: Z = 1.78 (P = 0.08)						
**2.4.2 Cohort**						
Chen et al. 2018	3	28	7	76	11.7%	1.16 [0.32, 4.19]
Kerleroux et al. 2020	26	130	6	42	28.9%	1.40 [0.62, 3.17]
Yoshimoto et al. 2021	4	49	13	108	16.9%	0.68 [0.23, 1.97]
**Subtotal (95% CI)**		**207**		**226**	**57.5%**	**1.09 [0.61, 1.94]**
Total events	33		26			
Heterogeneity: Tau² = 0.00; Chi² = 1.13, df = 2 (P = 0.57); I² = 0%						
Test for overall effect: Z = 0.29 (P = 0.77)						
**Total (95% CI)**		**716**		**727**	**100.0%**	**1.36 [0.88, 2.11]**
Total events	57		39			
Heterogeneity: Tau² = 0.00; Chi² = 3.85, df = 5 (P = 0.57); I² = 0%						
Test for overall effect: Z = 1.38 (P = 0.17)						
Test for subgroup differences: Chi² = 1.34, df = 1 (P = 0.25), I² = 25.4%						

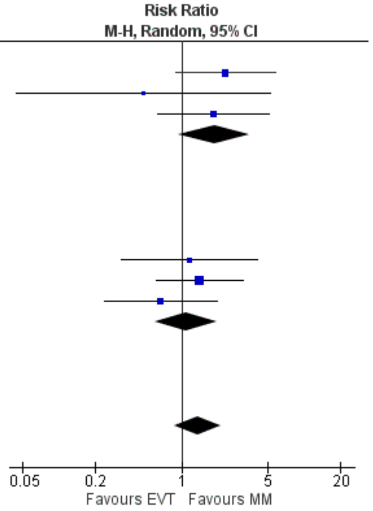

Figure S4. Mortality Rate, RCT vs. Cohort [8–11,24–28].

Study or Subgroup	EVT Events	EVT Total	MM Events	MM Total	Weight	Risk Ratio M-H, Random, 95% CI
**2.5.1 RCT**						
Huo et al. 2023	50	230	45	225	12.0%	1.09 [0.76, 1.56]
Huo et al. 2025	50	214	45	211	12.3%	1.10 [0.77, 1.56]
Sarraj et al. 2023	68	178	72	174	23.4%	0.92 [0.71, 1.19]
Thomalla et al. 2024	56	125	72	128	25.3%	0.80 [0.62, 1.02]
Yoshimura et al. 2022	18	101	24	102	5.2%	0.76 [0.44, 1.31]
**Subtotal (95% CI)**		**848**		**840**	**78.1%**	**0.92 [0.79, 1.05]**
Total events	242		258			
Heterogeneity: Tau² = 0.00; Chi² = 3.62, df = 4 (P = 0.46); I² = 0%						
Test for overall effect: Z = 1.24 (P = 0.22)						
**2.5.2 Cohort**						
Chen et al. 2018	6	28	26	76	2.6%	0.63 [0.29, 1.36]
Han et al. 2024	32	75	30	56	12.1%	0.80 [0.56, 1.14]
Kerleroux et al. 2020	41	130	13	42	5.8%	1.02 [0.61, 1.71]
Yoshimoto et al. 2021	4	49	18	108	1.5%	0.49 [0.17, 1.37]
**Subtotal (95% CI)**		**282**		**282**	**21.9%**	**0.80 [0.61, 1.04]**
Total events	83		87			
Heterogeneity: Tau² = 0.00; Chi² = 2.14, df = 3 (P = 0.54); I² = 0%						
Test for overall effect: Z = 1.65 (P = 0.10)						
**Total (95% CI)**		**1130**		**1122**	**100.0%**	**0.89 [0.78, 1.01]**
Total events	325		345			
Heterogeneity: Tau² = 0.00; Chi² = 6.43, df = 8 (P = 0.60); I² = 0%						
Test for overall effect: Z = 1.86 (P = 0.06)						
Test for subgroup differences: Chi² = 0.77, df = 1 (P = 0.38), I² = 0%						

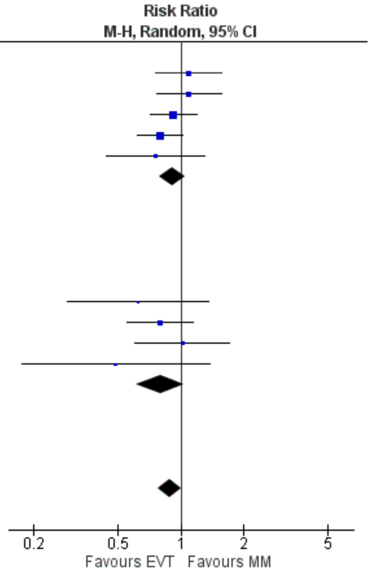

Figure S5. mRS at 3 Months (0–2), Asia vs. Europe [8–11,24–28].

	EVT		MM			Risk Ratio
**Study or Subgroup**	**Events**	**Total**	**Events**	**Total**	**Weight**	**M-H, Random, 95% CI**
**3.1.1 Asia**						
Chen et al. 2018	9	28	7	76	9.3%	3.49 [1.44, 8.48]
Han et al. 2024	15	75	5	56	8.6%	2.24 [0.87, 5.80]
Huo et al. 2023	69	230	26	225	14.9%	2.60 [1.72, 3.92]
Huo et al. 2025	63	214	23	211	14.6%	2.70 [1.74, 4.19]
Yoshimoto et al. 2021	21	49	3	108	6.9%	15.43 [4.83, 49.30]
Yoshimura et al. 2022	14	101	8	102	9.9%	1.77 [0.78, 4.03]
**Subtotal (95% CI)**		**697**		**778**	**64.3%**	**2.98 [1.99, 4.46]**
Total events	191		72			
Heterogeneity: Tau² = 0.12; Chi² = 10.18, df = 5 (P = 0.07); I² = 51%						
Test for overall effect: Z = 5.29 (P < 0.00001)						
**3.1.2 Europe**						
Kerleroux et al. 2020	30	130	11	42	12.6%	0.88 [0.49, 1.60]
Sarraj et al. 2023	36	178	12	174	12.3%	2.93 [1.58, 5.45]
Thomalla et al. 2024	28	125	8	128	10.8%	3.58 [1.70, 7.56]
**Subtotal (95% CI)**		**433**		**344**	**35.7%**	**2.07 [0.84, 5.06]**
Total events	94		31			
Heterogeneity: Tau² = 0.51; Chi² = 11.33, df = 2 (P = 0.003); I² = 82%						
Test for overall effect: Z = 1.59 (P = 0.11)						
**Total (95% CI)**		**1130**		**1122**	**100.0%**	**2.64 [1.79, 3.90]**
Total events	285		103			
Heterogeneity: Tau² = 0.22; Chi² = 24.03, df = 8 (P = 0.002); I² = 67%						
Test for overall effect: Z = 4.92 (P < 0.00001)						
Test for subgroup differences: Chi² = 0.53, df = 1 (P = 0.47), I² = 0%						

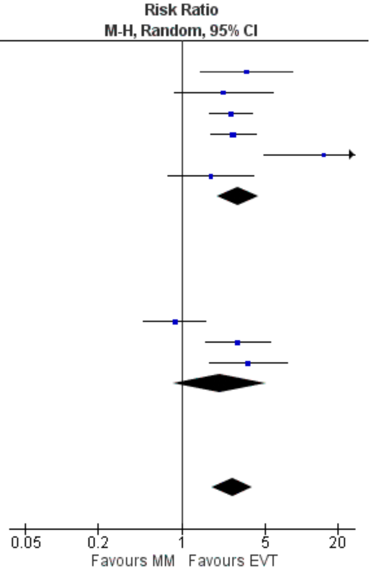

Figure S6. mRS at 3 Months (0–3), Asia vs. Europe [8–11,24–28].

Study or Subgroup	EVT Events	EVT Total	MM Events	MM Total	Weight	Risk Ratio M-H, Random, 95% CI
**3.2.1 Asia**						
Chen et al. 2018	11	28	14	76	7.7%	2.13 [1.10, 4.13]
Han et al. 2024	22	75	11	56	8.0%	1.49 [0.79, 2.82]
Huo et al. 2023	108	230	75	225	15.9%	1.41 [1.12, 1.77]
Huo et al. 2025	97	214	68	211	15.6%	1.41 [1.10, 1.80]
Yoshimoto et al. 2021	24	49	10	108	7.7%	5.29 [2.75, 10.19]
Yoshimura et al. 2022	31	101	13	102	8.8%	2.41 [1.34, 4.33]
**Subtotal (95% CI)**		**697**		**778**	**63.8%**	**1.92 [1.38, 2.67]**
Total events	293		191			
Heterogeneity: Tau² = 0.11; Chi² = 17.81, df = 5 (P = 0.003); I² = 72%						
Test for overall effect: Z = 3.87 (P = 0.0001)						
**3.2.2 Europe**						
Kerleroux et al. 2020	56	130	17	42	11.9%	1.06 [0.70, 1.61]
Sarraj et al. 2023	67	178	33	174	13.1%	1.98 [1.38, 2.85]
Thomalla et al. 2024	42	125	22	128	11.2%	1.95 [1.24, 3.08]
**Subtotal (95% CI)**		**433**		**344**	**36.2%**	**1.61 [1.07, 2.41]**
Total events	165		72			
Heterogeneity: Tau² = 0.08; Chi² = 5.87, df = 2 (P = 0.05); I² = 66%						
Test for overall effect: Z = 2.30 (P = 0.02)						
**Total (95% CI)**		**1130**		**1122**	**100.0%**	**1.78 [1.40, 2.26]**
Total events	458		263			
Heterogeneity: Tau² = 0.08; Chi² = 23.70, df = 8 (P = 0.003); I² = 66%						
Test for overall effect: Z = 4.74 (P < 0.00001)						
Test for subgroup differences: Chi² = 0.44, df = 1 (P = 0.51), I² = 0%						

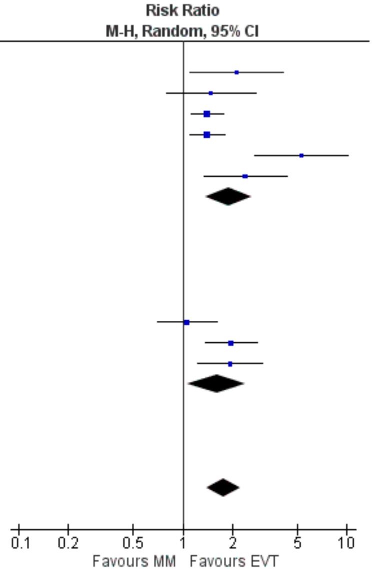

Figure S7. sICH Rate, Asia vs. Europe [8–10,26–28].

Study or Subgroup	EVT Events	EVT Total	MM Events	MM Total	Weight	Risk Ratio M-H, Random, 95% CI
**3.4.1 Asia**						
Chen et al. 2018	3	28	7	76	11.7%	1.16 [0.32, 4.19]
Huo et al. 2023	14	230	6	225	21.9%	2.28 [0.89, 5.83]
Yoshimoto et al. 2021	4	49	13	108	16.9%	0.68 [0.23, 1.97]
Yoshimura et al. 2022	9	101	5	102	17.2%	1.82 [0.63, 5.24]
**Subtotal (95% CI)**		**408**		**511**	**67.7%**	**1.41 [0.82, 2.44]**
Total events	30		31			
Heterogeneity: Tau² = 0.01; Chi² = 3.12, df = 3 (P = 0.37); I² = 4%						
Test for overall effect: Z = 1.24 (P = 0.21)						
**3.4.2 Europe**						
Kerleroux et al. 2020	26	130	6	42	28.9%	1.40 [0.62, 3.17]
Sarraj et al. 2023	1	178	2	174	3.4%	0.49 [0.04, 5.34]
**Subtotal (95% CI)**		**308**		**216**	**32.3%**	**1.25 [0.58, 2.72]**
Total events	27		8			
Heterogeneity: Tau² = 0.00; Chi² = 0.67, df = 1 (P = 0.41); I² = 0%						
Test for overall effect: Z = 0.57 (P = 0.57)						
**Total (95% CI)**		**716**		**727**	**100.0%**	**1.36 [0.88, 2.11]**
Total events	57		39			
Heterogeneity: Tau² = 0.00; Chi² = 3.85, df = 5 (P = 0.57); I² = 0%						
Test for overall effect: Z = 1.38 (P = 0.17)						
Test for subgroup differences: Chi² = 0.06, df = 1 (P = 0.80), I² = 0%						

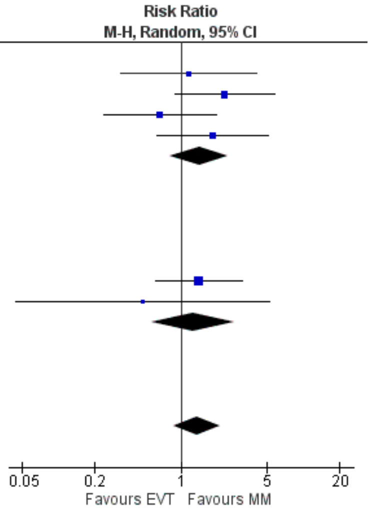

Figure S8. Mortality Rate, Asia vs. Europe [8–11,24–28].

	EVT		MM			Risk Ratio
Study or Subgroup	Events	Total	Events	Total	Weight	M-H, Random, 95% CI
**3.5.1 Asia**						
Chen et al. 2018	6	28	26	76	2.6%	0.63 [0.29, 1.36]
Han et al. 2024	32	75	30	56	12.1%	0.80 [0.56, 1.14]
Huo et al. 2023	50	230	45	225	12.0%	1.09 [0.76, 1.56]
Huo et al. 2025	50	214	45	211	12.3%	1.10 [0.77, 1.56]
Yoshimoto et al. 2021	4	49	18	108	1.5%	0.49 [0.17, 1.37]
Yoshimura et al. 2022	18	101	24	102	5.2%	0.76 [0.44, 1.31]
**Subtotal (95% CI)**		**697**		**778**	**45.6%**	**0.91 [0.75, 1.10]**
Total events	160		188			
Heterogeneity: Tau² = 0.00; Chi² = 5.24, df = 5 (P = 0.39); I² = 5%						
Test for overall effect: Z = 1.01 (P = 0.31)						
**3.5.2 Europe**						
Kerleroux et al. 2020	41	130	13	42	5.8%	1.02 [0.61, 1.71]
Sarraj et al. 2023	68	178	72	174	23.4%	0.92 [0.71, 1.19]
Thomalla et al. 2024	56	125	72	128	25.3%	0.80 [0.62, 1.02]
**Subtotal (95% CI)**		**433**		**344**	**54.4%**	**0.87 [0.74, 1.03]**
Total events	165		157			
Heterogeneity: Tau² = 0.00; Chi² = 1.06, df = 2 (P = 0.59); I² = 0%						
Test for overall effect: Z = 1.60 (P = 0.11)						
**Total (95% CI)**		**1130**		**1122**	**100.0%**	**0.89 [0.78, 1.01]**
Total events	325		345			
Heterogeneity: Tau² = 0.00; Chi² = 6.43, df = 8 (P = 0.60); I² = 0%						
Test for overall effect: Z = 1.86 (P = 0.06)						
Test for subgroup differences: Chi² = 0.09, df = 1 (P = 0.76), I² = 0%						

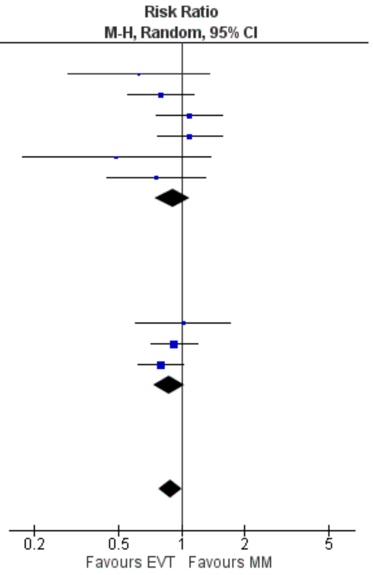

Figure S9. Sensitivity Analysis for mRS at 3 Months (0–2) Excluding Kerleroux et al., 2020 [8–11,24–28].

	EVT		MM			Risk Ratio
**Study or Subgroup**	**Events**	**Total**	**Events**	**Total**	**Weight**	**M-H, Random, 95% CI**
Chen et al. 2018	9	28	7	76	8.6%	3.49 [1.44, 8.48]
Han et al. 2024	15	75	5	56	7.7%	2.24 [0.87, 5.80]
Huo et al. 2023	69	230	26	225	22.2%	2.60 [1.72, 3.92]
Huo et al. 2025	63	214	23	211	21.0%	2.70 [1.74, 4.19]
Kerleroux et al. 2020	30	130	11	42	0.0%	0.88 [0.49, 1.60]
Sarraj et al. 2023	36	178	12	174	14.3%	2.93 [1.58, 5.45]
Thomalla et al. 2024	28	125	8	128	11.1%	3.58 [1.70, 7.56]
Yoshimoto et al. 2021	21	49	3	108	5.5%	15.43 [4.83, 49.30]
Yoshimura et al. 2022	14	101	8	102	9.6%	1.77 [0.78, 4.03]
**Total (95% CI)**		**1000**		**1080**	**100.0%**	**2.98 [2.22, 3.99]**
Total events	255		92			

Heterogeneity: $Tau^2 = 0.06$; $Chi^2 = 10.55$, df = 7 (P = 0.16); $I^2 = 34\%$
Test for overall effect: Z = 7.28 (P < 0.00001)

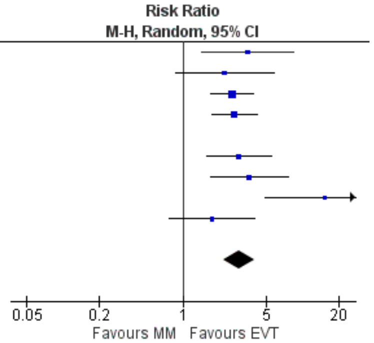

Figure S10. Sensitivity Analysis for mRS at 3 Months (0–3) Excluding Yoshimoto et al., 2021 [8–11,24–28].

	EVT		MM			Risk Ratio
**Study or Subgroup**	**Events**	**Total**	**Events**	**Total**	**Weight**	**M-H, Random, 95% CI**
Chen et al. 2018	11	28	14	76	5.6%	2.13 [1.10, 4.13]
Han et al. 2024	22	75	11	56	6.0%	1.49 [0.79, 2.82]
Huo et al. 2023	108	230	75	225	23.2%	1.41 [1.12, 1.77]
Huo et al. 2025	97	214	68	211	22.0%	1.41 [1.10, 1.80]
Kerleroux et al. 2020	56	130	17	42	11.7%	1.06 [0.70, 1.61]
Sarraj et al. 2023	67	178	33	174	14.2%	1.98 [1.38, 2.85]
Thomalla et al. 2024	42	125	22	128	10.3%	1.95 [1.24, 3.08]
Yoshimoto et al. 2021	24	49	10	108	0.0%	5.29 [2.75, 10.19]
Yoshimura et al. 2022	31	101	13	102	6.9%	2.41 [1.34, 4.33]
**Total (95% CI)**		**1081**		**1014**	**100.0%**	**1.58 [1.33, 1.87]**
Total events	434		253			

Heterogeneity: Tau² = 0.02; Chi² = 10.39, df = 7 (P = 0.17); I² = 33%

Test for overall effect: Z = 5.30 (P < 0.00001)

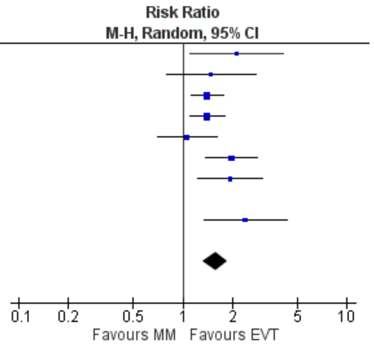

Figure S11. Sensitivity Analysis for Mean mRS Excluding Huo et al., 2023 [8–11,24].

	EVT			MM				Mean Difference
**Study or Subgroup**	**Mean**	**SD**	**Total**	**Mean**	**SD**	**Total**	**Weight**	**IV, Random, 95% CI**
Huo et al. 2023	3.67	1.5	230	4	1.1	225	0.0%	-0.33 [-0.57, -0.09]
Huo et al. 2025	3.67	2.2	214	4.33	1.65	211	24.9%	-0.66 [-1.03, -0.29]
Sarraj et al. 2023	4.33	1.65	178	5	1.1	174	39.7%	-0.67 [-0.96, -0.38]
Thomalla et al. 2024	4.66	1.65	125	5.32	1.1	128	28.3%	-0.66 [-1.01, -0.31]
Yoshimura et al. 2022	3.99	2.21	101	4.97	2.76	102	7.2%	-0.98 [-1.67, -0.29]
**Total (95% CI)**			**618**			**615**	**100.0%**	**-0.69 [-0.87, -0.50]**

Heterogeneity: Tau² = 0.00; Chi² = 0.75, df = 3 (P = 0.86); I² = 0%
Test for overall effect: Z = 7.31 (P < 0.00001)

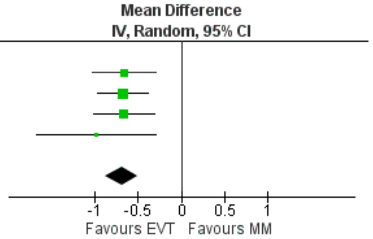

Supplement: Supplementary file 1 [file neurosci-07-00102-s001.zip › neurosci-4533960-supplementary.pdf]
